# Genetic diversity, evolution and selection in the major histocompatibility complex *DRB* and *DQB* loci in the family *Equidae*

**DOI:** 10.1186/s12864-020-07089-6

**Published:** 2020-09-30

**Authors:** Marie Klumplerova, Petra Splichalova, Jan Oppelt, Jan Futas, Aneta Kohutova, Petra Musilova, Svatava Kubickova, Roman Vodicka, Ludovic Orlando, Petr Horin

**Affiliations:** 1 Department of Animal Genetics, Veterinary and Pharmaceutical University, Brno, Czech Republic; 2 Ceitec VFU, RG Animal Immunogenomics, Brno, Czech Republic; 3 Ceitec MU, Masaryk University, Kamenice 753/5, 625 00 Brno, Czech Republic; 4 National Centre for Biomolecular research, Faculty of Science, Masaryk University, Kamenice 753/5, 625 00 Brno, Czech Republic; 5 Department of Biology, Faculty of Medicine, Masaryk University, Kamenice 753/5, 625 00 Brno, Czech Republic; 6 Department of Genetics and Reproductive Biotechnologies, Veterinary Research Institute, Brno, Czech Republic; 7 Ceitec VRI, RG Animal Cytogenomics, Brno, Czech Republic; 8 Zoo Prague, U Trojského zámku 120/3, 171 00 Praha 7, Czech Republic; 9 Laboratoire d’Anthropobiologie Moléculaire et d’Imagerie de Synthèse, CNRS UMR 5288, Université de Toulouse, Université Paul Sabatier, 31000 Toulouse, France; 10 Centre for GeoGenetics, Natural History Museum of Denmark, Øster Voldgade 5-7, 1350K, Copenhagen, Denmark

**Keywords:** Major histocompatibility complex, Family *Equidae*, MHC exon 2, MHC class II loci, Positive selection, Trans-species polymorphism, Selected amino acid sites

## Abstract

**Background:**

The mammalian Major Histocompatibility Complex (MHC) is a genetic region containing highly polymorphic genes with immunological functions. MHC class I and class II genes encode antigen-presenting molecules expressed on the cell surface. The MHC class II sub-region contains genes expressed in antigen presenting cells. The antigen binding site is encoded by the second exon of genes encoding antigen presenting molecules. The exon 2 sequences of these MHC genes have evolved under the selective pressure of pathogens. Interspecific differences can be observed in the class II sub-region. The family *Equidae* includes a variety of domesticated, and free-ranging species inhabiting a range of habitats exposed to different pathogens and represents a model for studying this important part of the immunogenome. While equine MHC class II *DRA* and *DQA* loci have received attention, the genetic diversity and effects of selection on *DRB* and *DQB* loci have been largely overlooked. This study aimed to provide the first in-depth analysis of the MHC class II *DRB* and *DQB* loci in the *Equidae* family.

**Results:**

Three *DRB* and two *DQB* genes were identified in the genomes of all equids. The genes *DRB2*, *DRB3* and *DQB3* showed high sequence conservation, while polymorphisms were more frequent at *DRB1* and *DQB1* across all species analyzed. *DQB2* was not found in the genome of the Asiatic asses *Equus hemionus kulan* and *E. h. onager*. The bioinformatic analysis of non-zero-coverage-bases of *DRB* and *DQB* genes in 14 equine individual genomes revealed differences among individual genes. Evidence for recombination was found for *DRB1*, *DRB2, DQB1* and *DQB2* genes. Trans-species allele sharing was identified in all genes except *DRB1*. Site-specific selection analysis predicted genes evolving under positive selection both at *DRB* and *DQB* loci. No selected amino acid sites were identified in *DQB3*.

**Conclusions:**

The organization of the MHC class II sub-region of equids is similar across all species of the family. Genomic sequences, along with phylogenetic trees suggesting effects of selection as well as trans-species polymorphism support the contention that pathogen-driven positive selection has shaped the MHC class II *DRB*/*DQB* sub-regions in the *Equidae*.

## Background

The mammalian Major Histocompatibility Complex (MHC) is a large genetic region of approximately 4 Mb containing about 230 protein coding genes, many of them encoding molecules with immunological functions. The ancestral MHC was composed of genes encoding molecules presenting antigens to T lymphocytes [1]. In eutherian mammals, genes coding for antigen presenting molecules are clustered into two regions, class I and class II, separated by a class III region encoding molecules not involved in the process of antigen presentation [2, 3]. The MHC class II region is a complex region containing different sub-regions with variable numbers of often highly polymorphic genes. Class II molecules coding for antigen presenting molecules are heterodimeric glycoproteins consisting of an α and a β chain encoded by MHC class II genes *DRA/DQA* and *DRB/DQB*, respectively. Class II molecules are expressed on the surface of antigen presenting cells where they interact with the T cell receptor (TCR) on CD4^+^ T lymphocytes. The antigen binding site (ABS) encoded by the exon 2 of the corresponding genes is located in a groove created by both α and β chains. It has the capacity to upload antigenic peptides of extracellular origin and present them to T lymphocytes [4]. The exon 2 sequences of the MHC loci encoding antigen-presenting molecules likely evolved under strong selection pressure of pathogens [5].

In eutherian mammals, the overall genomic organization of the MHC region is similar across different families. However, important interspecific differences can be observed in the organization of the class II region. The *DR* and *DQ* class II sub-regions were identified in almost all species hitherto studied [6, 7]. Typically, these regions contain at least one α chain and one β chain gene [8]. Gene duplications, losses and various intragenic mutations have resulted in variable numbers of genes and pseudogenes across mammals [9].

The family *Equidae* includes a variety of domesticated, free-ranging, and captive species inhabiting a wide range of habitats, showing different environmental and pathogenic exposure. It thus represents a suitable model for studying the immunogenome [10], including the MHC [11, 12]. Although the equine phylogenetic tree has remained debated for a long time [13, 14], recent phylogenomic work based on whole genome sequence data has revealed that the most recent common ancestor of non-caballine equids (zebras and asses) and caballine equids (domestic and wild horses) dates back to 4.0–4.5 Mya [15]. Within non-caballines, ass and zebra clades emerged some 1.69–1.99 Mya, with each clade diversifying into a number of individual species soon after [16]. The family *Equidae* seems to have evolved rapidly both at the molecular [17] and karyotypic level [18].

Like in other species, including humans, the equine MHC has originally been identified serologically as the “ELA” (Equine Leucocyte Antigen) complex [19]. According to the nomenclature suggested by Klein et al. [20], its current designation is *Eqca*. Despite the general importance of the domestic horse, its MHC has still not been fully characterized. Gustafson et al. [21] provided data on the genomic organization of the equine MHC genes by analyzing contigs spanning the MHC region. The horse full genome sequence assembly EquCab3.0 [22] provided a picture on the general organization of the equine MHC, based on in silico annotations. However, the annotation of this individual horse genome sequence as well as individual genomes of other equids did not resolve individual variation of the MHC region, especially in the class I and class II sub-regions coding for antigen-presenting molecules [23].

Viluma et al. [24] characterized the genomic structure of the horse MHC class II sub-region using long-read sequencing of bacterial artificial chromosomes (BAC) clones derived from a single stallion related to the donor of the reference genome sequence EquCab3.0. Besides non-classical class II genes and pseudogenes, they identified potentially expressed classical class II genes: one *DRA*, three *DRB* and three *DQA/DQB* pairs. Miller et al. [25] confirmed the expression status of the *DR* and *DQ* genes and provided evidence about their haplotype variation. At the population level, the genetic diversity of selected MHC class II loci has been studied in horses, donkeys and zebras [26–32].

The MHC loci are known to be among the most polymorphic loci in vertebrate genomes [4]. The generation and long-term maintenance of their polymorphism is believed to be driven by pathogens through balancing selection [33]. Balancing selection can be explained by heterozygote advantage, frequency-dependence or by fluctuating selection [34–37]. Effects of positive selection can be investigated by analyzing nucleotide sequences of the loci of interest. High levels of polymorphism, higher rates of non-synonymous (*d*_*N*_) to synonymous (*d*_*S*_) nucleotide substitutions and trans-species allele sharing are considered to be common features of balancing selection [35, 38–41].

Although the genetic diversity and effects of selection on MHC class II *DRA* and *DQA* loci have been studied in equids [11, 12, 42], little is known about equine *DRB* and *DQB* loci in this context. The objectives of this work were to study the nucleotide sequence diversity of exon 2 of the MHC class II *DRB* and *DQB* loci in the family *Equidae*, and to assess signatures of positive and negative selection on this functionally important domain of the antigen-presenting MHC class II molecules. Additionally, we used *TNFA*, a member of the MHC class III coding for the tumor-necrosis factor alpha, for comparison. This molecule is not involved in antigen presentation. Due to its crucial function in immunity, it is highly conserved among different mammalian species, including the horse [43].

## Results

### Identification of individual MHC *DRB* and *DQB* genes in equid genomes

**Fig. 1:**
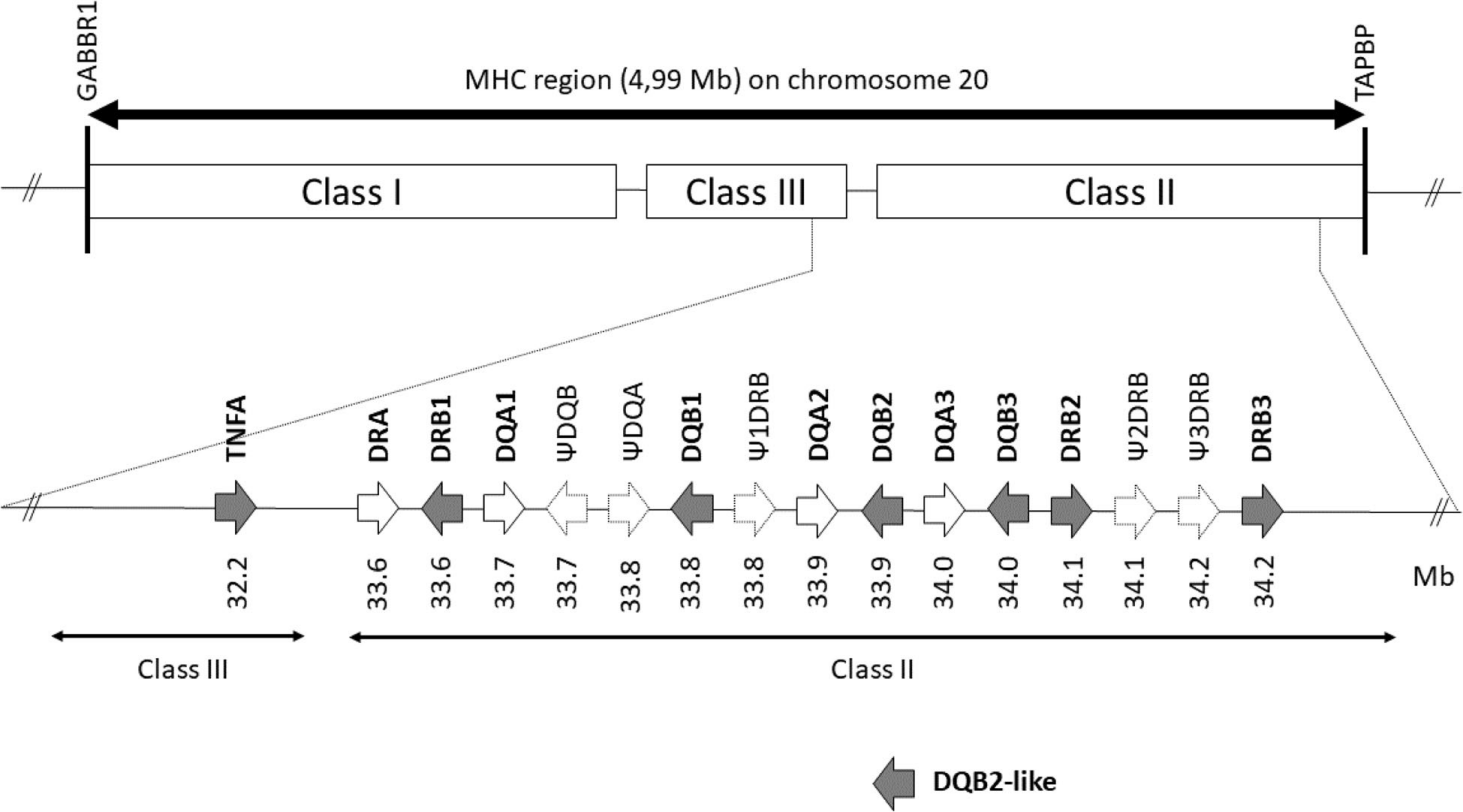
The horse *MHC DR* and *DQ* loci studied. Expressed *DR* and *DQ* genes located on ECA20 of EquCab3.0 are represented as solid-line arrows, pseudogenes as dashed-line arrows. Transcriptional directionality is indicated by arrows. Genes analyzed in this study are in bold. Based on Viluma et al. [24]

Leveraging on re-sequencing data and computational analyses of previously published genomic resources allowed us to identify three potentially functional *DRB* and three *DQB* genes with undisrupted open reading frames in equids. Sequences identified as pseudogenes and identical to those reported by Viluma et al. [24] were disregarded in the analyses presented below (see Fig. 1).

The potentially functional genes were numbered based on their position on the equine physical map, *DRB1-DRB3* and *DQB1-DQB3*, which corresponds to numbering by Viluma et al. [24]. A 5.5 kb long interspersed nuclear element (LINE/L1) found within the *DQB2* of EquCab 3.0, was also identified in the alignment sequence data underlying three individual horse genomes (Arabian, Standardbred and Norwegian Fjord horse) [15, 16] as well as in the de novo assemblies of the Przewalski’s horse (Burgud) [44] and the donkey ASM130575v1 (Maral har) [45].

A *DQB*-like sequence [46], not present in the reference genome EquCab3.0 nor in the de novo donkey genome assemblies ASM303372v1 (*E. asinus*) and ASM130575v1 (*E. asinus*) [45], was identified in de novo assembled genomes of Ajinai1.0 (Mongolian domestic horse) [44] and Burgud (*E. ferus przewalskii*) [44]. A comparison of its flanking regions found in the Burgud genome and the reference genome EquCab3.0 showed that the downstream flanking sequence from Burgud was highly similar (99% sequence identity) to a *DQB2* downstream flanking sequence from EquCab3.0. An upstream *DQB*-like flanking sequence from the Burgud genome showed intermediate (78%) similarity to the relevant EquCab3.0 *DQB2* upstream flanking sequence. Corresponding upstream and downstream flanking regions identified in the Ajinai1.0 assembly showed intermediate similarity (79–82%) to various sequences of the EquCab3.0 MHC class II region, with no specificity for *DQB2*. Sequence identities between the *DQB-*like gene upstream and downstream flanking sequences identified in Ajinai and Burgud assemblies were 90 and 81%, with 46 and 26% of the sequence covered, respectively. Pairwise sequence alignments of all flanking sequences analyzed are shown in Additional file 1. The FISH probe targeting the *DQB*-like sequence mapped to horse chromosome 20q21.1-q21.2 within the MHC (ELA) region. Hybridization signals were observed on both copies of chromosome 20 in horses positive as well as negative for the *DQB2*-like sequence as assessed by PCR. This precluded us from determining the locus/allele status of this sequence. Therefore, it was given a provisional designation “*DQB2-like*”. No paired signal was ever detected on any other chromosome region in the PCR positive horse.

### MHC *DRB* and *DQB* loci in the genomes of the *Equidae*

The bioinformatic analysis of non-zero-coverage-bases of *DRB* and *DQB* loci in 14 equine individual genomes revealed differences among individual genes (Table 1). The genes *DRB2*, *DRB3* and *DQB3* showed little differences between the genomes analyzed, with a very high percentage of non-zero-coverage-bases (> 80%). For the genes *DRB1* and *DQB1*, a lower percentage of non-zero-coverage-bases was observed across all species analyzed. The *DQB2* gene showed large differences among the genomes analyzed (Table 1), with very low values found within *E. ferus przewalskii*, *E. quagga burchellii* and *E. hemionus onager* genomes, suggesting that the gene was absent from the genomes of these individuals. Intermediate to high values were found for *E. grevyi, E. zebra hartmannae, E. asinus asinus, E. africanus somaliensis* and *E. kiang*. A variation of the non-zero coverage values was also observed within the domestic horse group. In the genomes of three horses (Arabian, Standardbred and Norwegian Fjord horse), the values for *DQB2* were high, suggesting that the gene was present. In contrast, the genomes of the Middle Pleistocene, Late Pleistocene and Icelandic horses exhibited low values, despite sequencing efforts to higher depth in the latter two.

**Table 1:** Percentage of non-zero-coverage bases of *DRB* and *DQB* loci among equid genomes. Values close to zero indicate absence of the sequence. Non-zero-coverage lower than 10% is shown in **bold**

	*DRB1*	*DRB2*	*DRB3*	*DQB1*	*DQB2*	*DQB3*
*E. caballus* (Late Pleistocene horse)	67	98	96	63	**4**	98
*E. caballus* (Middle Pleistocene horse)	30	81	80	39	**2**	92
*E. caballus* (Arabian)	35	96	90	52	57	98
*E. caballus* (Standardbred)	35	95	95	59	74	97
*E. caballus* (Norwegian Fjord horse)	55	94	94	43	83	94
*E. caballus* (Icelandic)	60	91	94	43	**3**	95
*E. ferus przewalskii*	36	95	94	49	**2**	93
*E. burchellii*	54	97	98	72	**9**	99
*E. grevyii*	49	99	93	51	93	97
*E. zebra hartmannae*	41	97	98	56	93	100
*E. asinus asinus*	48	92	98	55	94	88
*E. africanus somaliensis*	39	94	99	55	90	99
*E. hemionus onager*	46	95	99	61	**3**	100
*E. kiang*	47	98	98	65	79	96

PCR amplifications with different combinations of primer pairs also showed differences between and within species (Table 2 and Additional file 2). Individual PCRs with primer pairs for the *DRB1, DQB1, and DQB2* genes failed in some individuals (Additional file 2). For *E. quagga borensis* and *E. kiang*, it happened that *DRB1* and *DRB3* sequences retrieved from the same individual were identical, while *DQB* sequences obtained from the same individual following amplification of different genes were never identical. No sequence assigned to a given gene was identical to any sequence from another *DQB* gene across the entire family. The LINE/L1 element was found within the *DQB2* gene sequences in individual genomes of *E. grevyi, E. zebra hartmannae, E. asinus asinus, E. africanus somaliensis* and *E. kiang*.

**Table 2:** Numbers of exon 2 nucleotide sequences identified in individual equid species and sub-species

Species/Sub-species	No. of individuals	No. of exon 2 sequences (No. of sequences shared by at least two species/sub-species)						
		*DRB1*	*DRB2*	*DRB3*	*DQB1*	*DQB2*	*DQB2-like*	*DQB3*
*E. caballus*	11	2	6 (1)	3	5	5 (1)	1	4 (2)
*E. ferus przewalskii*	3	0	2 (1)	0	1 (1)	2 (1)	0	4 (3)
*E. grevyi*	2	1	1	1	1 (1)	1	0	2 (1)
*E. zebra hartmannae*	2	0	1 (1)	1 (1)	1 (1)	1	1 (1)	1 (1)
*E. quagga burchellii*	2	1	1 (1)	2	2	1	1 (1)	3 (2)
*E. quagga boehmi*	2	1	1	1	1	1	0	1 (1)
*E. quagga chapmani*	2	1	1	2 (1)	2 (1)	1	2	2 (1)
*E. quagga borensis*	2	1	2	1 (1)	1 (1)	1	1	2 (1)
*E. asinus asinus*	2	1	1	2	0	1	0	1 (1)
*E. africanus somaliensis*	2	1	2	2	1	3	2	3 (2)
*E. kiang*	2	2	1	1	2	1	1 (1)	3 (2)
*E. hemionus kulan*	2	1	2	3	2 (1)	0	2	2
No. of sequences (No. of sequences shared by at least two species/sub-species)		12 (0)	19 (2)	15 (1)	14 (1)	16 (1)	9 (1)	13 (4)
No. of amino acid sequences (No. of sequences with stop codon / frame-shift deletion)		12 (0/0)	19 (2/0)	14 (0/0)	14 (2/1)	16 (0/0)	9 (0/0)	10 (0/0)

A synopsis of data obtained by in silico analysis of non-zero coverage, PCR amplifications and *DQB2*-specific sequences retrieved in this study provided no evidence for the presence of this gene in Icelandic horses, while it could be identified in other breeds, both by in silico and molecular approaches. The gene could not be found in the whole genome sequences of a Przewalski’s horse and of a Burchell’s zebra, but PCR amplifications were successful for some other individuals of these species, and matched sequences of *DQB2* exon 2. No such comparisons could be made for *E. hemionus* asses. No DNA from onagers was available for molecular analysis, while no individual whole genome sequence was available for kulan at the time of the study. We note, however, that no *DQB2* sequence was found in the onager genome and no *DQB2* sequence could be retrieved from two kulans analyzed, despite the identification of two different *DQB2-like* sequences in this species, one of which was shared in *E. q. burchelli* and in some Przewalski’s horses.

### Allelic diversity of exon 2 sequences: polymorphism at *DRB* and *DQB* loci

The alignment of exon 2 sequences assigned to all individual *DRB* and *DQB* loci is in Additional file 3. The total numbers of *DRB* and *DQB* sequences retrieved in this study, along with the corresponding numbers of alleles identified, the numbers of alleles shared between species, are summarized in Table 2. In the entire family, we identified a total of 44 exon 2 *DRB* sequences, of which three were shared by at least two species (GenBank accession numbers MF997084 - MF997132). Two exon 2 sequences (Eqbu-DRB1*0401/Eqbu-DRB3*0101 and Eqki-DRB1*0201/Eqki-DRB3*0101) were identical between the *DRB1* and *DRB3* genes in the same species. Altogether 52 exon 2 *DQB* nucleotide sequences (GenBank accession numbers MF997133 - MF997201) were identified across the entire family, seven of which were shared by at least two species. Thirty-eight exon 2 *DRB* and 46 exon 2 *DQB* sequences are novel sequences. All non-caballine equine sequences obtained were novel.

Allele sharing was identified in all genes except *DRB1*. In *DQB3*, one allele was shared by all the species analyzed except *Equus hemionus*. All *DRB* and *DQB* nucleotide sequences code for 43 and 52 amino acid sequences, respectively. Two sequences with a stop-codon were found at each *DRB2* and *DQB1* gene in the domestic and Przewalski’s horse. A frame-shift deletion was found in *Equus kiang* in the *DQB1* sequence (summarized in Table 2). A three-bp long deletion was found in one *DQB1* sequence in the horse, in seven *DQB2* sequences in five species, including *E. grevyi, E. zebra hartmannae, E. burchellii, E. kiang,* and *E. caballus,* and in two *DQB3* sequences in *E. ferus przewalskii* and *E. caballus*.

Standard diversity indices calculated for each gene for the entire family *Equidae* documenting differences in diversity between MHC class II loci are provided in Table 3. They include sequences obtained in this study along with sequences available in GenBank [47] and in the IPD database [48].

**Table 3:** Standard diversity indices and global-selection at individual genes and at sub-region-level

Locus/sub-region	Length (bp)	N	VNP	PIP	Z-test	
					p-value	d_N_-d_S_
*DQB1* locus	269	17	99	68	**0.033**	1.856
*DQB2* locus	269	19	92	46	0.062	1.550
*DQB3* locus	269	14	13	3	1.000	−0.610
*DQB2-like* sequence	269	9	32	24	1.000	−0.156
*DQB* sub-region	269	59	141	104	**0.042**	1.747
*DRB1* locus	260	16	93	67	1.000	−0.303
*DRB2* locus	260	20	63	37	0.157	1.012
*DRB3* locus	260	15	40	11	1.000	−0.204
*DRB* sub-region	260	52	114	84	0.375	0.318

*N* numbers of sequences, *VNP* variable nucleotide positions, *PIP* parsimony informative positions. Z-test p-value: probability of rejecting the null hypothesis of strict-neutrality (dN = dS) in favor of dN > dS. Significant *p*-values are in **bold**

### Phylogenetic and selection analyses

Phylogenetic trees obtained for exon 2 *DRB* and *DQB* sequences are shown in Additional files 4 and 5, respectively. The trees showed that in some cases, namely for the *DQB3* clade, alleles from the same loci, but different species, formed well supported clades. However, bootstrapping in the *DQB* and especially the *DRB* tree were generally weak to make conclusions about effects of selection on the sequences analyzed.

Due to a high conservation of the *TNFA* coding sequences analyzed, their phylogenetic tree was constructed for full-length sequences. Two clades supported by bootstrap pseudoreplicate values of 71 and 81% were identified (Additional file 6). One clade contained all zebras, the other one all asses and the domestic horse. Within the first clade, individual zebra species formed monophyletic clusters with basal *E. grevyi* and with sister clusters of *E. zebra hartmannae* and *E. quagga*. This is not reminiscent with the tree constructed by Jonsson et al. [16] based on genome-wide sequence data, in which *E. grevyi* and *E. quagga* appeared as sister species. Evidence for recombination events was found for the *DRB1*, *DRB2, DQB1*, and *DQB2* genes, as documented in Additional file 7.

The Z-test performed across all codon sites was statistically significant at the *DQB1* gene (*p*-value = 0.033) and at the *DQB* sub-region-level (*p*-value = 0.042), allowing the rejection of the null hypothesis of neutral evolution (d_N_ = d_S_) in favor of the alternative hypothesis of positive selection (d_N_ > d_S_). No evidence of positive selection at the level of the complete exon 2 sequences was found for other genes/sub-regions analyzed (Table 3).

Numbers of sites predicted to be under positive/diversifying selection in *DRB* and *DQB* are shown in Table 4. Most selected amino acid sites (SAASs) were observed in genes *DRB1, DQB1,* and *DQB*2 (Table 4). Within the coding sequence of the *TNFA* gene, one positively (position 178) and three negatively (positions 46, 224, 234) selected amino acid sites were identified.

**Table 4:** Numbers of amino acid sites under positive and negative selection identified in MHC *DRB* and *DQB* exon 2 sequences in equids

Selection	Positive		Negative	
Locus/sub-region	*DRB*	*DQB*	*DRB*	*DQB*
*1*	11	9	7	5
*2*	6	7	3	7
*3*	1	0	4	4
*Sub-region*	11	15	15	13

### Analysis of functional effects of amino acid substitutions

All non-synonymous variants were identified as tolerated by the SIFT software. PROVEAN and PolyPhen identified different deleterious and/or possibly/probably damaging variants, respectively (Additional file 8). Altogether 24 amino acid substitutions were identified as deleterious and/or possibly/probably damaging by both software tools (Table 5). A total of 11 of these amino acid substitutions were located in the putative ABS of the respective molecules. Among these 11 substitutions, 5 were found in selected amino acid sites. Reciprocally, all 5 five SAASs were located in the ABS (Tables 5, 6).

**Table 5:** Substitutions identified as “Deleterious” and “Possibly damaging/Probably damaging” both by Provean and PolyPhen2, respectively

Gene	AA position	Substitution	ABS	SAAS
*DRB1*	15	G - > V	NO	NO
*DRB1*	42	Y - > V	NO	NO
*DRB1*	53	A - > P	NO	NO
*DRB1*	55	Y - > K	YES	NO
*DRB1*	72	T - > K	NO	NO
*DRB2*	23	D - > F	NO	NO
*DRB2*	25	Y - > D	YES	NO
*DRB2*	25	Y - > L	YES	NO
*DRB2*	51	P - > T	YES	YES
*DRB3*	15	G - > V	NO	NO
*DRB3*	56	W - > L	NO	NO
*DRB3*	73	Y - > V	YES	NO
*DQB1*	24	R - > T	NO	NO
*DQB1*	43	R - > L	NO	NO
*DQB1*	60	K - > D	NO	NO
*DQB2*	24	R - > S	NO	NO
*DQB2*	25	Y - > I	YES	YES
*DQB2*	67	R - > W	YES	NO
*DQB2-like*	49	G - > A	YES	NO
*DQB2-like*	51	P - > W	YES	YES
*DQB2-like*	51	P - > S	YES	YES
*DQB2-like*	51	P - > L	YES	YES
*DQB3*	10	C - > R	NO	NO
*DQB3*	53	A - > P	NO	NO

**Table 6:** Non-synonymous amino acid substitutions, antigen binding sites and selected amino acid sites within the MHC class II loci analyzed

Gene	Lenght (AA)	No of variants	No of ABS	No of SAAS
*DRB1*	86	68	36	28
*DRB2*	86	42	21	9
*DRB3*	86	25	11	1
*DQB1*	89	67	27	24
*DQB2*	89	43	15	13
*DQB2-like*	89	22	16	4
*DQB3*	89	7	1	0

## Discussion

The data on MHC *DRB* and *DQB* loci, along with our previous results obtained for *DRA* and *DQA* loci [11], provide information on genomic diversity, evolution and selection of this functionally important group of MHC class II genes in the family *Equidae.*

The MHC class II region has been recently characterized by using long read NGS (LR-NGS) of a single horse [24]. Although LR-NGS was not available at the time of this study, our data obtained by NGS, Sanger sequencing and bioinformatic analyses of individual genomes are in good agreement with those obtained by Viluma et al. [24] and by Miller et al. [25] both in terms of the functional loci identified and their chromosomal organization. Our study provides an independent cross-confirmation of these data, while it extends previous findings to a wider range of domestic horses, the feral Przewalski’s horse, and to other equid species.

Based on our comparisons of all assembled individual genome sequences, including de novo assembled whole genome sequences, it seems that the individual (Twilight) reference sequence is in some of its parts rather atypical. We have found the three previously identified *DRB* loci, *DRB1-DRB3* in all equine species. While *DRB2* and *DRB3* genomic sequences were highly similar across the whole family, low percentage of non-zero-coverage-bases showed that the sequence annotated in the reference genome EquCab3.0 as *DRB1* differed from *DRB1* sequences retrieved from all equids including other domestic horses (Table 1). The reference *DRB1* sequence thus seems to be rather rare among *DRB1* sequences identified so far in the entire family. It is not clear whether this may be explained by higher diversity of horse/equid *DRB1* sequences compared to other genes. Co-amplifications of certain *DRB1* and *DRB3* sequences may indicate limited locus-specificity of the PCR primers used or that the same exon 2 allelic sequence is maintained at two different genes. However, if this occurred on a regular basis, the numbers of similar sequences from different loci would be higher than those observed in our dataset.

The three *DQB* genes studied also correspond to those reported previously [24, 25]. Even in the tree with weak node support, most of the *DQB* sequences retrieved with the *DQB* primers used here clustered according to gene (Additional file 5), suggesting their high locus-specificity in all equids. When we used the same primers with DNA of horses previously analyzed by Matiasovic et al. [49], we were unable to retrieve all allelic sequences originally identified with locus non-specific primers (data not shown). It is thus likely that the presence of additional variants in these animals resulted in an allelic drop-out, and that additional *DQB* variability still exists, at least in horses.

It is difficult to use a hard sequence cut-off to predict the presence/absence of genes due to possible base compositional bias in the data. However, a combination of our bioinformatic approach and of sequencing data suggested the existence of a variation in the presence/absence of the *DQB2* gene among equid species as well as within *E. caballus*. The status of a *DQB2*–like sequence first reported by Mashima [46] remains unclear. Within-species individual variation in the presence/absence of this sequence was observed in domestic horses, Przewalski’s horses and in *E. quagga*, where sufficient numbers of individuals could be examined (Additional file 2, Table 2). Its presence in individual de novo assembled genomes of domestic and Przewalski’s horses [44] and its absence from the horse EquCab3.0 assembly as well as from the de novo donkey genome assemblies ASM303372v1 [50] and ASM130575v1 [45] suggest that failures in our amplification attempts are not due to allelic drop-outs. However, we cannot rule out that the bioinformatic procedures used for genome annotations failed to assign highly similar sequences to two different genes.

These findings could be interpreted as *DQB2* allelic variability and/or as the existence of an additional *DQB* gene. Neither sequences comparisons nor phylogenetic analyses allowed us to resolve this problem. The corresponding branches of the phylogenetic tree were only weakly supported. The values of similarities of *DQB2* and *DQB2-like* flanking sequences are different in different individual genomes of Mongolian and Przewalski’s horses and of EquCab3.0 (Additional file 1). However, it is not clear whether these differences are due to differences in the quality of different assemblies. As our FISH probe eventually proved to be non-specific for the sequence analyzed, it only revealed that the *DQB*-like sequences analyzed were not located outside the horse MHC, in contrast to situations reported for some mammalian species [51].

The data indicate that like in other vertebrates including different mammalian families, the genetic diversity of *DRB* and *DQB* genes in equids can be determined not only by allelic polymorphisms but also by variation in the number of genes between species as well as within species. The horse reference assembly is an individual genome exhibiting copy number variations (CNVs) compared to other horses both within and across breeds; the highest CNV numbers were identified on the chromosome 20 [52, 53]. Miller et al. [25] have shown the existence of CNV for the horse MHC class II *DQA3* gene. Variation in the number of *DQ* genes was also observed in cattle [54, 55].

As usual in most other species [56], our analysis of MHC allelic polymorphisms focused on exon 2 sequences encoding the functionally important antigen binding site. Combining NGS, standard Sanger sequencing, and cloning, we were able to resolve most of exon 2 haplotypes. In those species where sufficient numbers of individuals were available, i.e. in domestic horses and in *Equus quagga*, exon 2 sequences of all three *DRB* and/or *DQB* genes were found to be polymorphic. One sequence was retrieved for some genes in other species due probably to low numbers of individuals analyzed (two for other than equine species, see Table 2). 44 and 52 *DRB* and *DQB* exon 2 sequences, respectively, identified in the entire panel of equids are lower numbers compared to the most studied mammalian species [48]. It seems that an important part of the MHC class II variation of equids still remains overlooked, mainly due to limited access to relevant numbers of representatives of individual equine species. Since the *DRB* locus-specific primers do not amplify complete exon 2 sequences, the allelic variability in these loci is likely to be even higher than that detected with our primers.

Recombination within the *DRB* and *DQB* regions was identified for the *DRB1, DRB2, DQB1* and *DQB2* loci by the Single Breakpoint (SBP) method (Additional file 7). Similarly to other species, and in agreement with Viluma et al. [24], our data illustrate that recombination events also occurred in the evolution of both *DRB* and *DQB* loci in equids.

In terms of potential functional importance, all the loci analyzed here were shown to be expressed in the horse [25]. Therefore, they are likely to be subject to various selective pressures. In fact, evidence for positive and negative selection was found for both *DRB* and *DQB* genes by various methods (Additional file 9). On the other hand, bootstrapping in the *DQB* and especially the *DRB* tree was too weak to make conclusions about effects of selection on the sequences analyzed.

The evolutionary tree of the *TNFA* locus located between class III and class II regions approximately 1.5 Mb apart from the loci analyzed here showed only subtle differences from the recently established equid phylogeny [16]. Like in other species, these findings can be interpreted as effects of balancing selection supposed to maintain the MHC class II polymorphism [39, 57], and of purifying selection preserving the structure and functions of the TNFA molecule in immune responses [58]. Despite the close physical proximity of the two genomic sequences, the effects of selection are characteristic for the particular type of loci.

Mechanisms of concerted evolution based on interlocus recombination or gene conversion may have contributed to the diversity observed in these loci, which is in agreement with the results of our recombination analysis (Additional file 7), and it also might be an explanation for identical exon 2 sequences occasionally found for two different MHC class II loci in the same species. The sequence data retrieved here suggest that a birth-and-death process might be a more important mechanism generating MHC polymorphism in equids, similarly to human and mouse MHC class II genes [52].

Trans-species polymorphism (TSP) manifested by trans-species sharing of allelic lineages is a typical feature of the MHC polymorphism, also interpreted as a consequence of positive selection [59]. We have observed trans-species allele sharing in all genes except *DRB1* (Additional file 10). Along with data from other vertebrate species, it supports the view that shared allelic lineages have special adaptive value and have been maintained by selection [60]. In this context, extinct Pleistocene horses represent an interesting source of information [15]. Although MHC sequences extracted from the genome of the Late Pleistocene horse (#CGG10022) are consensus sequences and not directly determined haplotypes, most of them were identical to allelic haplotype sequences observed in recent horses. Alleles shared across species can be inherited from a common ancestor and maintained independently by means of balancing selection, or they can indicate adaptive introgression, as shown in hominins. As it seems that some specific allelic lineages have persisted in equids over long time, the former interpretation is more plausible for this particular family.

In this context, one of the *DQB3* alleles shared by all species analyzed, except *Equus hemionus,* deserves special attention. It is this particular sequence that was found in the Late Pleistocene horse re-sequencing data. However, the reason for this long-term persistence is not clear. No signs of positive selection and only weak effects of purifying selection were identified within the *DQB3* gene. The *DQB3* sequences also showed much lower value of variable nucleotide positions as compared to *DQB1* and *DQB2* genes. Therefore, we can speculate that this conserved allele may bind and present some conserved epitope(s), and that its permanent selective pressure maintained this particular allelic sequence over time.

As the power of selection analyses strongly depends on the numbers of sequences analyzed, the data presented are not a complete list of all loci and/or amino acid sites under selection, but they point out sites under the strongest selection pressures. For the same reason probably, cumulative effects of selection could be detected over sub-regions rather than for individual genes.

The intensity of selection expressed as numbers of positively selected sites varied among individual genes but it was comparable between *DRB* and *DQB* loci. It was stronger for *beta*-loci (*DRB*, *DQB*) analyzed here compared to *alpha*-loci (*DRA*, *DQA*) analyzed previously, where 1 and 7 selected amino acid sites (SAASs) were identified, respectively [11]. Stronger selective pressure in *beta*-loci was described in grey wolves [61] and giant panda [62] as well. This may be related to differences in the level of sequence polymorphism in each gene [61], which we also have observed for our *DRA/DQA* vs. *DRB/DQB* comparisons. We may assume that both the polymorphism and the selected sites identified are caused by balancing selection.

Selected amino acid sites were detected both in the putative ABS residues and outside. The majority of SAASs identified in equids were also identified as SAASs in other species. Eleven out of 26 and 16 out of 21 SAAS identified within *DRB* and *DQB* loci respectively were shared with at least one species (Additional file 11). Interestingly, evidence for diversifying selection was observed for the ABS of the putative DQB2-like molecule (Additional file 9), which suggests that this molecule and its polymorphism might be of functional importance.

These findings are also supported by bioinformatic analyses of functional impacts of the amino acid changes found in functionally important sites of the molecules analyzed. According to the SIFT software, all non-synonymous substitution may be tolerated; on the other hand, an important proportion (46%) of changes assigned as potentially damaging both by PROVEAN and PolyPhen were located in the ABS, with five of them identified as SAASs. An overall interpretation of these findings could be that the non-synonymous substitutions analyzed can potentially change the function of the respective protein. However, since the software tools used were designed primarily for analyzing proteins not subject to positive/diversifying selection, we could assume that the non-synonymous substitutions, located especially in the ABS and subject to positive selection, are not neutral, but not necessarily damaging. Their location and the selection analysis suggest that they could be even beneficial.

We also could detect a weak signal of positive selection within the conservative *TNFA* gene. The extent to which this could be due to positive selection or to strong linkage disequilibrium with antigen presenting loci or to other reasons, and how far it can be related to the tree constructed by Jónsson et al. [16] based on genome-wide sequence data remains unknown. Effects of negative selection could be detected in all genes analyzed (Additional file 9). Taking into consideration constrains related to the functional importance of the molecules under study, this is not a surprising finding.

## Conclusions

Mammalian MHC class II loci show a high rate of adaptive evolution. Although the *Equidae* appears as a rapidly-evolving mammalian family, the organization of their MHC class II sub-region is highly similar across all species. Genomic sequences and trans-species polymorphism support the assumption that pathogen-driven positive selection has formatted the MHC class II *DRB*/*DQB* sub-regions in the *Equidae*. Information generated in this study can be further used for a more accurate annotation of the MHC region in all equine species, for studying mechanisms of evolution of the immunogenome as a result of host and pathogen interactions and consequently for studying the genetic basis of various diseases.

## Methods

### Animals

Blood samples of *E. ferus przewalskii, E. asinus asinus, E. africanus somaliensis, E. kiang, E. hemionus kulan, E. quagga burchellii, E. quagga boehmi, E. quagga chapmanni, E. quagga borensis, E. grevyi* and *E. zebra hartmannae* were obtained from The Prague Zoo (Dr. Roman Vodička) and Zoo Dvůr Králové, Czech Republic (Dr. Jiří Vahala). Samples of both the grey and black varieties of the Old Kladruby horses were collected at the National Stud of Kladruby nad Labem, Czech Republic. Samples of English Thoroughbred horses were collected at the Stud of Napajedla, Czech Republic, of Czech Warmbloods at the Teaching farm of the University of Veterinary and Pharmaceutical Sciences Brno, Nový Jičín, Czech Republic. Blood samples of Arab and Akhal-Teke originated from private farms (unnamed) in Chrastava, Czech Republic. Blood samples of Camargue horses were collected in the Camargue region, France and shared by Dr. Agnes Leblond, VetAgro Sup, Lyon, France. The Welsh Pony samples were collected at a private farm, Ranch Ladna, Czech Republic. DNA samples of Icelandic horses originating from private owners in Switzerland were shared by dr. Eliane Marti, University of Berne, Switzerland. Blood samples of Murgese horses originating from farms in Reggio di Puglia, Italy were shared by Dr. Ingrid Alloggio, University of Bari, Italy. Blood samples of Mongolian and Romanian horses originating from individually owned horses in Mongolia and in the Danube delta, Romania, respectively, were shared by Dr. David Modry, University of Veterinary and Pharmaceutical Sciences Brno, Czech Republic. The blood sample used for cytogenetic analyses was obtained from a horse housed at the Horse clinic, University of Veterinary and Pharmaceutical Sciences Brno, Czech Republic.

### Identification of MHC class II genes

Individual *DRB* and *DQB* genes were BLAST searched in the de novo assembled horse and donkey genomes Ajinai1.0 (*E. caballus*, Mongolian), Burgud (*E. ferus przewalskii)* [44], ASM303372v1 (*E. asinus asinus*) [50] and ASM130575v1 (*E. asinus asinus*) [45], using default parameters and the full-length gene sequences derived from the reference genome EquCab3.0 (coordinates in Table 7). A sequence of a *DQB* gene (GenBank accession: AB106863.2) described by Mashima [46], absent from the reference genome EquCab3.0 was searched following the same procedure. Flanking sequences were identified, compared with the reference genome EquCab3.0, allowing direct assignment to individual genes.

**Table 7:** Position of genes analyzed in the reference genome EquCab3.0, primer sequences and PCR annealing temperatures

Gene	Position of the gene analyzed (EquCab3.0)	Strand	Amplification details	Forward primer	Reverse primer	Annealing T (°C)	PCR product lenght (bp)
TNFA	32,223,398.. 32,226,182	+	TNFA_5UTR	CCTTTCAGAAGACCCATCCA	CATCTCGGATCATGCTTTCA	59.9	777
			TNFA_1CR	TAAACAGCCAGGCGATTTTCTCCCT	CCTACAACATGGGCTACAGGCTTG	57.5	1144
			TNFA_2CR	TGCCTTCCAGTCAATCAACCCTCT	GGTCACACATCCCTGCATTCTAGGTT	61.5	1192
			TNFA_3UTR	TGAGCCCATCTACCTGGGAGGAGT	GCAGAGGTTCAGCGATGTAGCGA	59	868
*DRB1*	33,625,487.. 33,631,729	–	1st round	GGGACGTGTTTAAGATGGGT	AACCACACACCCTCTCCACTG	7x(62–0,3/cycle) followed by 60	812
			2nd round	TGACCGGATCCTTCCTGTAC	GCGCTCACCTCGCCGAC	60	303
*DRB2*	34,096,675.. 34,108,525	+	1st round	TGTCCTTCAGGTGGAGGCAA	TCACACACTGACAACCACACATT	65	793
			2nd round	TGACCCGATCSTTCCTGTAT	RCGCTCACCTCGCCGAG	13x(65–0,3/cycle) followed by 61	303
*DRB3*	34,266,651.. 34,285,281	+	1st round	ACTCGCTCACAGTCCTACACAC	GTGCTGGTAGTTCGTGCGTGG	65	532
			2nd round	TGACCGGATCCTTCCTGTAC	GCGCTCACCTCGCCGAT	13x(65–0,3/cycle) followed by 61	303
*DQB1*	33,812,679.. 33,820,407	–		CCTCTGGGGTAACGTTCCAG	CGGCCTTGCTTTAGGTTTATC	4x(63–0,5/cycle) followed by 61	590
*DQB2*	33,941,932.. 33,956,144	*–*		AGGTTTCTCCCACTCAACTGCCTGA	GGACGCGCCCACCTCCCTGTCC	66	522
*DQB3*	34,031,398.. 34,037,071	*–*		AGGTTTATCCGATCCAACCGGCTGC	GCCCTCCCAGCTCCGAGACT	4X(68–0,5/cycle) followed by 66	451
*DQB2-like*	unknown	unknown		GCTCTCCTGGCGCAGAGACT	ACAGGGCTCTCATTTCCTTGTA	65.5	603
				GGTCAGAGCGGGAGGCGAGT	GCCCCATAAGCTTCGCAGCA	64	902

The sequences of the *DRB* and *DQB* loci identified were sought in the alignment sequence data underlying 14 individual equine genomes: a Late Pleistocene horse, a Middle Pleistocene horse, an Arabian, a Standardbred, an Icelandic, a Norwegian and a Fjord horse, as well as one member of each of the following 8 taxonomic groups (*E. ferus przewalskii*, *E. asinus asinus*, *E. africanus somaliensis*, *E. kiang*, *E. hemionus onager*, *E. quagga burchelli*, *E. grevyi* and *E. zebra hartmannae*) [15, 16]. The Paleomix pipeline [63] with stringent conditions and frequent validations ensuring high-quality alignments [64] was used for aligning the next generation sequencing (NGS) generated reads to the reference genomes. The coverage of individual *DRB* and *DQB* genes were determined. The percentage of non-zero-coverage-bases detecting captured regions of the sequence under study was calculated as the proportion of bases covered by at least one read out of the entire sequence studied. Non-covered regions may be interpreted as missing from the genome or undetected by our protocols [65]. Here, we arbitrarily set the threshold indicating the absence of a genomic region from our sequences as 90% zero-covered bases.

Five individual animals of Przewalski’s horse (*E. ferus przewalskii*) and five individuals from each of the following 12 domestic horse breeds (*E. caballus*) were used for studying within-species polymorphism of selected genes: grey and black variety of Old Kladruby horses, English Thoroughbred, Czech Warmblood, Arabian, Akhal-Teke, Murgese, Camargue, Welsh Pony, Icelandic, Jordanian, Mongolian and Romanian horses. Exon 2 sequences of all individual genes were amplified with the locus-specific primers as described below, and the presence/absence of PCR products was assessed by gel electrophoresis.

The nomenclature suggested by Klein et al. [20], and implemented previously by us for the *Equidae* [11] was used everywhere in the text.

### Next generation sequencing of bacterial artificial chromosomes

Eight equine bacterial artificial chromosomes (BACs): CH241-73 L13, CH241-72F8, CH241-326G2, CH241-441 N13, CH241-211I10, CH241-367G2, CH241-389G9, CH241-169B13, spanning a selected sub-region of the class II region (chr20: 33,562,617- 33,896,623) in EquCab3.0 were chosen from the NCBI clone database [66] and provided from the CHORI-241 equine BAC library (Children’s Hospital Oakland Research Institute, BACPAC Resources, Oakland, USA). BACs were sequenced by using Roche GS Junior following the manufacturer’s Rapid Library Preparation Protocol. Basic data processing was done by the GS Run Processor application, followed by data analysis with the GS De Novo Assembler. The contigs obtained were analyzed in a BLAST-search [67] against the reference genome.

### Cytogenetic analyses: fluorescence in situ hybridization (FISH)

To assess the so far unclear annotation of a MHC class II sequence originally reported by Mashima [46], which was not assigned to any known locus, genomic DNA of a horse previously identified as carrier of the sequence analyzed was amplified using the following primers: DQB-probeF (5′-AAGGCCCAGTGCTACTTCAC-3′) and DQB-probeR (5′-CCAGTCACCAGTCATAATAGTC-3′). The total reaction volume of PCR was 12.5 μl, consisting of 2.5 μl 5x KAPA2G Buffer A, 2.5 μl 5x KAPA Enhancer 1, 0.25 μl 10 mM KAPA dNTP mix, 0.625 μl of each 10 μM primer, 0.1 μl KAPA2G Robust DNA Polymerase (KAPA2G Robust HotStart PCR Kit, KAPABIOSYSTEMS, Cape Town, South Africa) and 0.5 μl of template. The cycling parameters included initial denaturation at 95 °C for 3 min, 34 cycles of 30 s denaturation at 95 °C, 30 s annealing at 64 °C and 2.5 min extension at 72 °C, followed by final extension at 72 °C for 3 min. The PCR product obtained was purified using High Pure PCR Purification Kit (Roche, Mannheim, Germany) and cloned into the pDrive Cloning Vector (Qiagen) and the recombinant plasmids were labelled with biotin-16-dUTP (Roche Diagnostics GmbH, Mannheim, Germany) by the Nick Translation Reagent Kit (Vysis, Richmond, UK). The labelled probes were used for a standard fluorescence in situ hybridization (FISH) to horse metaphase chromosomes prepared from peripheral blood of horses previously recognized as positive and negative for this sequence by means of PCR amplification. Sites of hybridization were visualized by immunodetection using Cy3-avidin (Amersham, Arlington Heights, IL, USA). Metaphase cells were captured using a Zeiss Axio Imager 2 fluorescence microscope equipped with appropriate filters and the slide scanning system Metafer (MetaSystems, Altlussheim, Germany) and analyzed using the ISIS software (MetaSystems).

### Amplification of selected *DRB* and *DQB* genes

Based on the reference whole genome sequence, we designed horse locus-specific primers for a number of genes. The locus-specificity in *DRB* genes that are highly similar to each other was ensured using a nested-PCR protocol. Primers for the second PCR amplification were designed based on sequences obtained by the first PCR amplification. Since in this case the reverse primer (2nd round of nested PCR) must be located in the exon-intron boundaries, nine nucleotides of the exon 2 sequence are missing in the *DRB* exon 2 sequences generated, while for all *DQB* genes, complete exon 2 sequences were obtained. For *DRB*, mismatches were introduced to reach the required locus specificity. The primer sequences and annealing temperatures, PCR product lengths and positions of the genes amplified are shown in Table 7. These horse primers were then also used for amplifying the corresponding exon 2 sequences in other equids.

Samples of genomic DNA from at least two individual animals of 12 taxonomic groups - *Equus caballus* (*n* = 11)*, E. ferus przewalskii* (*n* = 3)*, E. asinus asinus* (*n* = 2)*, E. africanus somaliensis* (*n* = 2)*, E. kiang* (*n* = 2)*, E. hemionus kulan* (*n* = 2)*, E. quagga burchellii* (*n* = 2)*, E. quagga boehmi* (*n* = 2)*, E. quagga chapmanni* (*n* = 2)*, E. quagga borensis* (*n* = 2)*, E. grevyi* (*n* = 2)*,* and *E. zebra hartmannae* (*n* = 2)*, −* were available for sequencing.

Genomic DNA extraction was performed using a NucleoSpin Blood kit (Macherey-Nagel, Duren, Germany) from EDTA-treated peripheral blood according to the manufacturer’s instructions. The total reaction volume of PCR was 12.5 μl, consisting of 2.5 μl 5xHerculase II buffer, 0.125 μl dNTP mix (25 mM each dNTP), 0.313 μl of each 10 μM primer, 0.125 μl s Herculase II Fusion DNA Polymerase (Agilent technologies, Santa Clara, CA, United States) and 0.5 μl of template. The cycling parameters included initial denaturation at 95 °C for 2 min, 30 cycles of 20 s denaturation at 95 °C, 20 s annealing according to Table 7 and 30 s extension at 72 °C, followed by final extension at 72 °C for 3 min.

### Amplification of the *TNFA* gene

The complete *Tumor necrosis factor alpha* (*TNFA)* gene was amplified in four separate PCRs using primers and annealing temperatures as described in Table 7.

### PCR amplicon sequencing

All PCR products were sequenced by standard Sanger sequencing (Macrogen, Inc., Seoul, South Korea). Putative heterozygotes (Sanger sequences containing double peaks) were further analyzed using the Roche GS FLX+ sequencing platform according to manufacturer’s Universal tailed amplicon sequencing design. Original primers were modified by adding a universal tail, which served as a priming site for a second PCR round in which MID sequences were introduced in the PCR product in order to enable post-sequencing individual sequence de-multiplexing. Products were mixed equimolarly and sequenced at Eurofins MWG operon (Ebersberg, Germany). All sequenced reads were sorted according to their MID tags, the MID tags were clipped. Reads corresponding to individual MID tags were further sorted based on the primer sequences using the CD-HIT suite [68] and a 90% identity cut-off. Primer sequences were trimmed with Cutadapt [69]. The reads obtained were further clustered by using the CD-HIT suite with the 0.99 sequence identity cut-off and cluster’s consensual sequences were manually compared with the corresponding Sanger sequences in Bioedit [70].

### Molecular cloning

Molecular cloning was used to resolve double peaks in Sanger sequences and/or to confirm sequences obtained from amplicon sequencing in certain cases. Corresponding PCR products were cloned with the CloneJet™ PCR cloning kit (Fermentas) and the TransformAid Bacterial Transformation Kit (Thermo Scientific) according to manufacturer’s instructions. Individual colonies were transferred directly into the PCR mix. DNA was extracted during initial denaturation and served as a template for amplification with primers given in Table 7. The PCR product obtained was sequenced by standard Sanger sequencing. Maximum of 6 colonies were used for PCR amplifications and sequencing.

### Sequence alignment

The sequences retrieved were aligned by using the MAFFT alignment software [71]. For the *DRB* and *DQB* loci, primer and/or intronic sequences were trimmed. For selection analyses, sequences were trimmed to match the open reading frame. For *TNFA*, the whole gene sequence (including introns and untranslated regions) and the coding sequence were determined. For the purposes of selection analyses, stop codon nucleotides were removed.

### Determination of allelic exon 2 sequences

Allelic sequence variants were identified based on next generation and Sanger sequencing of PCR amplicons obtained with locus-specific primers and/or of the clones produced. A sequence was considered as an allelic haplotype only if it was retrieved from at least two independent PCRs*.* For individual genomes sequenced originally for other purposes [15, 16], we used original BAM files for extracting haplotype sequences. Other de novo assembled genomes were also searched for exon 2 allelic sequences at the loci analyzed. If the sequence retrieved matched a sequence identified by other methods, it was considered for haplotype validation. Altogether, each allele was identified based on two independent sources of information on its haplotype sequence. All other sequences were discarded from analyses of allelic diversity.

Variable nucleotide positions and parsimony informative positions were calculated for each gene for all *Equidae* using MEGA6 [72].

### Analysis of selection

Selection analyses were carried out at the level of individual genes and at the sub-region level. Sequences obtained in this study along with sequences available in GenBank [47] and in the IPD database [48] assigned to specific genes were used for the analysis of individual genes [15, 16]. All available *DRB*/*DQB* sequences were then used for the analysis at the sub-region-level. Phylogenetic trees were constructed to compare them with trees based on selected evolutionarily neutral genes.

Maximum likelihood phylogenetic trees were constructed using MEGA6 [72] and tested with 1000 Bootstrap replications. All trees were inferred by using the Nearest-Neighbor-Interchange and NJ/BioNJ as an initial tree. Sequences from GenBank [47] *Ovis aries* (KC733431), *Bos taurus* (HQ199077), *Sus scrofa* (AY135575) and *Ovis aries* (Z28424), *Bos taurus* (AY444376), *Sus scrofa* (JQ511975) were used as outgroup sequences for *DRB* and *DQB* phylogenetic analyses, respectively. Sequences derived from individual reference genomes of *Sus scrofa* (NC_010449.4 and JF831365, respectively), *Bos taurus* (NC_007324.5 and NM_173966.3) and *Ovis aries* (NC_019477.1 and NM_001024860.1) were used as outgroups for phylogenetic analyses of the *TNFA* sequences. Different substitution models were analyzed and the model with the lowest BIC score was chosen. Information of the substitution model used for the phylogeny reconstruction can be found in the description of individual phylogenetic trees.

All alignments were screened for recombination events prior the site-specific selection analyses. The evidence for recombination was based on the single breakpoint recombination method (SBR) implemented in Datamonkey web server [73]. The small sample AIC (cAIC) was used as a default criterion for the decision on the presence of recombination. Recombination breakpoints were considered in further selection analyses; the data were analyzed in two partitions.

The global selection averaged across all amino acid sites was estimated by using the Z-test implemented in MEGA6 [72] by comparing the relative rates of non-synonymous (d_N_) and synonymous (d_S_) substitutions with the Jukes and Cantor correction. The alternative hypothesis of non-neutrality was tested with the probability of rejecting the null hypothesis of strict-neutrality (d_N_ = d_S_) in favor of the alternative hypothesis of positive selection (d_N_ > d_S_).

Detection of amino acid sites under selection was performed using the Datamonkey web server [73]. An automatic model selection tool provided by Datamonkey was used individually for each alignment to estimate the nucleotide evolution model. Selection was detected by codon-based maximum likelihood methods [74] and the FUBAR method [75]. For comparison, the MEME method [76] for identifying sites under episodic positive selection, was used. Taking into consideration the low numbers of sequences available for individual genes, SLAC and FEL methods were used for the analysis of genomic sub-regions only. Potential recombination breakpoints were taken into consideration.

Site-specific selection was also identified by using the CodeML algorithm within the PAML v4.3 package [77]. Different ω (dN/dS) parameters were estimated under four codon substitution models (M1a – nearly neutral, M2a – positive selection, M7 – no positive selection and M8 – positive selection). We compared the fit of the models with and without selection by the likelihood ratio test. Posterior probabilities for positively selected sites in models M2a and M8 were inferred by the Bayes empirical Bayes approach.

Codons identified by at least two of the previously mentioned methods were considered to be under positive selection. Predictions of putative antigen binding sites (ABS) were based on the human and murine MHC class II molecule structure [78, 79].

### Analysis of functional effects of amino acid substitutions

Consequences of amino acid substitution were analyzed with Provean [80], SIFT [81] and PolyPhen [82] software using homologies and sequence alignments to predict whether a change in amino acid sequence has a potential damaging effect on the protein function. Analyses were carried out for individual genes. Variants corresponding to the reference genome EquCab 3.0 were used as query sequences (not applicable to *DQB2-like* gene). All non-synonymous substitutions were analyzed with SIFT and PROVEAN software. Standard threshold 0.05 was used for the SIFT analysis. For PROVEAN analysis, the threshold was set up to − 4.5 to ensure higher specificity. The non-synonymous substitutions detected as “deleterious” by PROVEAN were additionally analyzed with PolyPhen.

## Supplementary information

**Additional file 1. **BLAST pairwise sequence alignments of *DQB2* and *DQB-like* flanking sequences.

**Additional file 2. **Amplification of individual *DRB* and *DQB* genes from different horse breeds.

**Additional file 3. **Alignment of *DRB* and *DQB* sequences analyzed.

**Additional file 4.** Maximum likelihood phylogeny reconstruction of all unique DRB alleles.

**Additional file 5.** Maximum likelihood phylogeny reconstruction of all unique DQB alleles. The tree was inferred using the Jukes-Cantor model with discrete Gamma distribution and tested by 1000 Bootstrap replications. The tree is drawn to scale, with branch lengths measured in the number of substitutions per site. Sequences, which were not obtained in this study are in brackets.

**Additional file 6.** Maximum likelihood phylogeny reconstruction of TNFA gene.

**Additional file 7.** Recombination identified by the Single breakpoint (SBP) method.

**Additional file 8.** Functional effects of amino acid substitutions.

**Additional file 9. **Positively and negatively selected sites identified using different methods in *DRB* and *DQB* loci.

**Additional file 10. **Allele sharing in individual *DRB* and *DQB* loci.

**Additional file 11. **Sharing of positively selected amino acid sites (SAASs) in the *DRB* and *DQB* locus in Equids and other species.

## Data Availability

Complete datasets used and/or analyzed during this study are available from the corresponding author upon a reasonable request. All data presented are publically available. Individual sequences can be downloaded from GenBank (https://www.ncbi.nlm.nih.gov/genbank/) under accession numbers MF997084 - MF997132 (*DRB* loci), MF997133 - MF997201 (*DQB* loci), and MG029639 - MG029658 (*TNFA*), KC733431, HQ199077, AY135575, Z28424, AY444376, JQ511975, NC_010449.4, JF831365, NC_007324.5, NM_173966.3, NC_019477.1, NM_001024860.1 (outgroup sequences), AB106863.2 (*DQB* sequence). De novo assembled horse and donkey genomes can be found at https://www.ncbi.nlm.nih.gov/assembly/ under accession numbers GCA_002863925.1 (EquCab3.0), GCA_000696655.1 (Ajinai1.0), GCA_000696695.1 (Burgud), GCA_003033725.1 (ASM303372v1, *E. asinus asinus*), GCA_001305755.1 (ASM130575v1, *E. asinus asinus*). Equine genomes are available in the Sequence Read Archive (https://www.ncbi.nlm.nih.gov/sra) under the accession number SRA082086.

## Abbreviations

BAC: Bacterial artificial chromosomes
MHC: Major histocompatibility complex
PCR: Polymerase chain reaction
SAAS: Selected amino acid site
TNFA: Tumor necrosis factor alpha
