# Supplementary material for: Genetic diversity, evolution and selection in the major histocompatibility complex DRB and DQB loci in the family Equidae
Source: BMC Genomics. 2020 Sep 30;21:677. doi: 10.1186/s12864-020-07089-6 (PMC7525986; doi:10.1186/s12864-020-07089-6)
Supplement: Supplementary file 3 — Additional file 3. Alignment of DRB and DQB sequences analyzed. [file 12864_2020_7089_MOESM3_ESM.pdf]

Alignment of *DRB* and *DQB* sequences analyzed. Sequences in parenthesis were not obtained in this study.

```
                           10        20        30        40        50
                   ....|....|....|....|....|....|....|....|....|....|
Eqca-DRB1*00101    CACATTTCCTGGAGGCTGTGAAGTTCGAGTGTCGTTTCTCCAACGGGACT
(Eqca-DRB1*00201)  CACATTTCCTGGAGGCTGTGAAGTACGAGTGTCATTTCTCCAACGGGACT
(Eqca-DRB1*00301)  CACATTTCCTGGAGCTGGTTAAACACGAGTGTCATTTCTCCAACGGGACT
(Eqca-DRB1*00401)  CACATTTCCTGGAGTATACTACGTCCGAGTGTCATTTCTCCAACGGGACT
(Eqca-DRB1*00501)  CTCATTTCCTGGAGCTGGTTAAACACGAGTGTCATTTCTCCAACGGGACT
Eqca-DRB1*0601     CACATTTCCTGCAGTATCTTAAGTTCGAGTGTCATTTCTCCAACGGGACT
Eqgr-DRB1*0101     CACATTTCCTGGAGTATCTTAAGTTCGAGTGTCATTTCTCCAACGGGACT
Eqbu-DRB1*0101     CACATTTCCTGGAGTATCTTAAGTTCGAGTGTCATTTCTCCAACGGGACT
Eqbu-DRB1*0201     CACATTTTCTGGAGTATGCGAAGGCCGAGTGTCATTTCTCCAACGGGACG
Eqbu-DRB1*0301     CACATTTTCTGGAGTATGCGAAGGCCGAGTGTCATTTCTCCAACGGGACG
Eqas-DRB1*0101     CACATTTCCTGGGGTATACTACGTCCGAGTGTCATTTCTCCAACGGGACT
Eqbu-DRB1*0401     CACATTTCCTGGAGTATAGTACCTTCGAGTGTCATTTCTCCAACGGGACT
Eqas-DRB1*0201     CACATTTCCTGGAGTATCTTAAGTTCGAGTGTCATTTCTCCAACGGGACT
Eqhe-DRB1*0101     CACATTTCCTGGAGTATCTTAAGTTCGAGTGTCATTTCTCCAACGGGACT
Eqki-DRB1*0101     CACATTTCCTGGAGTATCTTAAGTTCGAGTGTCATTTCTCCAACGGGACT
Eqki-DRB1*0201     CACATTTCCTGGAGTATAGTACCTTCGAGTGTCATTTCTCCAACGTGACT
Eqca-DRB2*00101    CACATTTCCTGGAGCTGGTTAAACACGAGTGTCATTTCTCCAACGGGACT
Eqca-DRB2*00201    CACATTTCCTGGAGCTGGTTAAACACGAGTGTCATTTCTCCAACGGGACT
Eqca-DRB2*00301    CACATTTCCTGGAGATGGTTAAACACGAGTGTCATTTCTCCAACGGGACT
(Eqca-DRB2*00401)  CACATTTCCTGGAGTATAGTACGTCCGAGTGTCATTTCTCCAACGGGACT
Eqca-DRB2*0501     CACATTTCCTGGAGCTGGTTAAACACGAGTGTCATTTCTCCAACGGGACT
Eqca-DRB2*0601     CACATTTCCTGGAGTATAGTACGTCCGAGTGTCATTTCTCCAACGGGACT
Eqca-DRB2*0701     CACATTTCCTGGAGCTGGTTAAACACGAGTGTCATTTCTCCAACGGGACT
Eqpr-DRB2*0101     CACATTTCCTGGAGCTGGTTAAACACGAGTGTCATTTCTCCAACGGGACT
Eqpr-DRB2*0201     CACATTTCCTGGAGCTGGTTAAACACGAGTGTCATTTCTCCAACGGGACT
Eqgr-DRB2*0101     CACATTTCCTGGAGTATAGTACGTCCGAGTGTCATTTCTCCAACGGGACT
Eqze-DRB2*0101     CACATTTCCTGGAGTATAGTACGTCCGAGTGTCATTTCTCCAACGGGACT
Eqbu-DRB2*0101     CACATTTCCTGGAGTATAGTACGTCCGAGTGTCATTTCTCCAACGGGACT
Eqbu-DRB2*0201     CACATTTCCTGGAGCTGGTTAAACACGAGTGTCATTTCTCCAACGGGACT
Eqbu-DRB2*0301     CACATTTCCTGGAGTATAGTACTTTCGAGTGTCATTTCTCCAACGGGACT
Eqbu-DRB2*0401     CACATTTCCTGGAGCTGGTTAAACACGAGTGTCATTTCTCCAACGGGACT
Eqbu-DRB2*0501     CACATTTCCTGGAGCTGGTTAAACACGAGTGTCATTTCTCCAACGGGACT
Eqas-DRB2*0101     CACATTTCCTGGAGTATAGTACGTCCGAGTGTCATTTCTCCAACGGGACT
Eqas-DRB2*0201     CACATTTCCTGGAGCTGGTTAAACACGAGTGTCATTTCTCCAATGGGACG
Eqas-DRB2*0301     CACATTTCCTGGAGCTGGTTAAACACGAGTGTCATTTCTCCAACGGGACT
Eqhe-DRB2*0101     CACATTTCCTGGAGTATAGTACGTCCGAGTGTCATTTCTCCAACGGGACT
Eqhe-DRB2*0201     CACATTTCCTGGAGTATAGTACGTCCGAGTGTCATTTCTCCAACGGGACT
Eqki-DRB2*0101     CACATTTCCTGGAGTATAGTACGTCCGAGTGTCATTTCTCCAACGGGACT
Eqca-DRB3*00101    CACATTTCCTGGAGTATAGTACGTCCGAGTGTCATTTCTTCAACGGAACA
Eqca-DRB3*00201    CACATTTCCTGGAGTATAGTACCTTCGAGTGTCATTTCTCCAACGGGACT
Eqca-DRB3*0301     CACGTTTCCTGGAGTATAGTACCTTCGAGTGTCATTTCTCCAACGGGACT
Eqgr-DRB3*0101     CACATTTCCTGGAGTATAGTACCTTCGAGTGTCATTTCTCCAACGGGACT
Eqze-DRB3*0101     CACATTTCCTGGAGTATAGTACCTTCGAGTGTCATTTCTCCAACGGGACT
Eqbu-DRB3*0101     CACATTTCCTGGAGTATAGTACCTTCGAGTGTCATTTCTCCAACGGGACT
Eqbu-DRB3*0201     CACATTTCCTGGAGTATAGTACCTTCGAGTGTCATTTCTCCAACGGGACT
Eqbu-DRB3*0301     CACATTTCCTGGAGTATAGTACCTTCGAGTGTCATTTCTCCAACGGGACT
Eqbu-DRB3*0401     CACATTTCCTGGAGTATAGTACCTTCGAGTGTCATTTCTCCAACGGGACT
Eqas-DRB3*0101     CACATTTCCTGGAGTATAGTACCTTCGAGTGTCATTTCTCCAACGGGACT
Eqas-DRB3*0201     CACATTTCCTGGAGTATAGTACCTTCGAGTGTCATTTCTCCAACGGGACT
Eqas-DRB3*0301     CACATTTCCTGGAGTATAGTACCTTCGAGTGTCATTTCTCCAACGGGACT
Eqhe-DRB3*0101     CACATTTCCTGGAGTATAGTACCTTCGAGTGTCATTTCTCCAACGGGACT
Eqhe-DRB3*0201     CACATTTCCTGGAGTATAGTACCTTCGAGTGTCATTTCTCCAACGGGACT
Eqhe-DRB3*0301     CACATTTCCTGGAGTATAGTACCTTCGAGTGTCATTTCTCCAACGTGACT
Eqki-DRB3*0101     CACATTTCCTGGAGTATAGTACCTTCGAGTGTCATTTCTCCAACGTGACT
```

```
                                60        70        80        90       100
                           ....|....|....|....|....|....|....|....|....|....|
Eqca-DRB1*00101            GAGCGAGTGCGATTCCTGGAGAGACGCTTCCATAACGGGGAGGAGTACGC
(Eqca-DRB1*00201)          GAGCGGGTGCGATACCTGGAAAGACTCTTCTATAACGGGAAGGAGTTCGT
(Eqca-DRB1*00301)          CAGCGGGTGCGGTTCCTGGACAGATACTTCCATAACGGGAAGGAGTTCGT
(Eqca-DRB1*00401)          CAGCGGGTGCGGTTCCTGGACAGATACTTCTATAACGGGAAGGAGACCGT
(Eqca-DRB1*00501)          CAGCGGGTGCGATTCCTGGACAGATACTTCTATAACAGGGAGGAGTACGT
Eqca-DRB1*0601             GAGAGGGTGAGATACCTAATGAGAATCTTCTATAACGGGGAGGAGAACGT
Eqgr-DRB1*0101             GAGAGGGTCCGATACCTGGTCAAAGTTTTCTATAACGGGGAGGAGTACGT
Eqbu-DRB1*0101             GAGAGGGTCCGATACCTGGTCAAAGTCTTCTATAACGGGGAGGAGTACGT
Eqbu-DRB1*0201             GAGCGGGTGCGGTTCCTGGCCAGAATCATCTACAACCAGGAGGAGTGGGT
Eqbu-DRB1*0301             GAGCGGGTGCGGTTCCTGGCCAGAGTCATCTACAACCAGGAGGAGTACGT
Eqas-DRB1*0101             CAGCGGGTGCGGTTCCTGGACAGATACTTCTATAACGGGAAGGAGTTCGT
Eqbu-DRB1*0401             CAGCGGGTGCTATACTTGCACAGACTCTTCTATAACGGGAAGGAGTACGT
Eqas-DRB1*0201             GAGAGGGTCCGATACCTGGTCAAAGTCTTCTATAACGGGGAGGAGTACGT
Eqhe-DRB1*0101             GAGAGGGTCCGATACCTGGTCAAAGTCTTCTATAACGGGGAGGAGTACGT
Eqki-DRB1*0101             GAGAGGGTCCGATACCTGGTCAAAGTCTTCTATAACGGGGAGGAGTACGT
Eqki-DRB1*0201             CAGCGGGTGCTATACTTGCACAGACTCTTCTATAACGGGAAGGAGAACGT
Eqca-DRB2*00101            CAGCGGGTGCGGTTCCTGGACAGATACTTCTATAACAGGGAGGAGTACGT
Eqca-DRB2*00201            CAGCGGGTGCGGTTCCTGGACAGATACTTCTATAACAGGGAGGAGTACGT
Eqca-DRB2*00301            CAGCGAGTGCGGTTCCTGGACAGATACTTCTCTAACGGGGAGGAGAACGT
(Eqca-DRB2*00401)          CAGCGAGTGCGGTTCCTGGACAGATACTTCTCTAACGGGGAGGAGACCGT
Eqca-DRB2*0501             CAGCGGGTGCGGTTCCTGGACAGATACTTCTATAACAGGGAGGAGTACGT
Eqca-DRB2*0601             CAGCGGGTGCGGTTCCTGGACAGATACTTCTATAACAGGGAGGAGTACGT
Eqca-DRB2*0701             CAGCGGGTGCGGTTCCTGGACAGATACTTCTATAACAGGGAGGAGTACGT
Eqpr-DRB2*0101             CAGCGGGTGCGGTTCCTGGACAGATACTTCTATAACAGGGAGGAGTACGT
Eqpr-DRB2*0201             CAGCGGGTGCGGTTCCTGGACAGATACTTCTATAACAGGGAGGAGTACGT
Eqgr-DRB2*0101             CAGCGAGTGCGGTTCCTGGACAGATACTTCTCTAACGGGGAGGAGAACGT
Eqze-DRB2*0101             CAGCGGGTGCGGTTCCTGGACAGATACTTCTATAACAGGGAGGAGTACGT
Eqbu-DRB2*0101             CAGCGGGTGCGGTTCCTGGACAGATACTTCTATAACAGGGAGGAGTACGT
Eqbu-DRB2*0201             CAGCGGGTGCGGCTCCTGGACAGATACTTCTATAACAGGGAGGAGTACGT
Eqbu-DRB2*0301             CAGCGGGTGCTATACTTGCACAGACTCTTCTATAACGGGAAGGAGTACGT
Eqbu-DRB2*0401             CAGCGAGTGCGGTTCCTGGACAGATACTTCTCTAACGGGGAGGAGAACGT
Eqbu-DRB2*0501             CAGCGGGTGCGGTTCCTGGACAGATACTTCTATAACAGGGAGGAGTACGT
Eqas-DRB2*0101             CAGCGGGTGCGGTTCCTGGACAGATACTTCTATAACAGGGAGGAGTACGT
Eqas-DRB2*0201             GAGAGGGTGCGGTATGTATTCAGAGACGTCTACAACCGGGAGGAGCACGT
Eqas-DRB2*0301             CAGCGGGTGCGGTTCCTGGACAGATACTTCTATAACAGGGAGGAGTACGT
Eqhe-DRB2*0101             CAGCGGGTGCGGTTCCTGGACAGATACTTCTATAACAGGGAGGAGTACGT
Eqhe-DRB2*0201             CAGCGGGTGCGGTTCCTGGACAGATACTTCTATAACAGGGAGGAGTACGT
Eqki-DRB2*0101             CAGCGGGTGCGGTTCCTGGACAGATACTTCTATAACAGGGAGGAGTACGT
Eqca-DRB3*00101            GAGCGAGTGCGGTACTTGGACAGATACTTCTATAACGGGAAGGAGTACGT
Eqca-DRB3*00201            CAGCGGGTGCTATACTTGCACAGACTCTTCTATAACGGGAAGGAGTACGT
Eqca-DRB3*0301             CAGCGGGTGCTATACTTGCACAGACTCTTCTATAACGGGAAGGAGTACGT
Eqgr-DRB3*0101             CAGCGGGTGCTATACTTGCACAGACTCTTCTATAACGGGAAGGAGTACGT
Eqze-DRB3*0101             CAGCGGGTGCTATACTTGCACAGACTCTTCTATAACGGGAAGGAGTACGT
Eqbu-DRB3*0101             CAGCGGGTGCTATACTTGCACAGACTCTTCTATAACGGGAAGGAGTACGT
Eqbu-DRB3*0201             CAGCGGGTGCTATACTTGCACAGACTCTTCTATAACGGGAAGGAGTACGT
Eqbu-DRB3*0301             CAGCGGGTGCTATACTTGCACAGACTCTTCTATAACGGGAAGGAGTACGT
Eqbu-DRB3*0401             CAGCGGGTGCTATATTTGCACAGACTCTTCTATAACGGGAAGGAGTACGT
Eqas-DRB3*0101             CAGCGGGTGCTATACTTGCACAGACTCTTCTATAACGGGAAGGAGTACGT
Eqas-DRB3*0201             CAGCGGGTGCTATACTTGCACAGACTCTTCTATAACGGGAAGGAGTACGT
Eqas-DRB3*0301             CAGCGGGTGCTATACTTGCACAAACTCTTCTATAACGGGAAGGAGTACGT
Eqhe-DRB3*0101             CAGCGGGTGCTATACTTGCACAGACTCTTCTATAACGGGAAGGAGTACGT
Eqhe-DRB3*0201             CAGCGGGTGCTATACTTGCACAGACTCTTCTATAACGGGAAGGAGTACGT
Eqhe-DRB3*0301             CAGCGGGTGCTATACTTGCACAGACTCTTCTATAACGGGAAGGAGTACGT
Eqki-DRB3*0101             CAGCGGGTGCTATACTTGCACAGACTCTTCTATAACGGGAAGGAGAACGT
```

```
                                   110       120       130       140       150
                          ....|....|....|....|....|....|....|....|....|....|
Eqca-DRB1*00101           GCGCTTCGACAGCGACGTGGGGGAGTACCGGGCGGTGACCGAGCTGGGGC
(Eqca-DRB1*00201)         GCGCTTCGACAGCGACGTGGGGGAGTTTCGGGCGGTGACCGAGCTGGGGC
(Eqca-DRB1*00301)         GCGCTTCGACAGCGACGTGGGGGAGTTCCGGGCGGTGACCGAGCTGGGGC
(Eqca-DRB1*00401)         GCGCTTCGACAGCGACGTGGGGGAGTACCGGGCGGTGACCGAGCTGGGGC
(Eqca-DRB1*00501)         GCGCTTCGACAGCGACGTGGGGGAGTACCGGGCGGTGACCGAGCTGGGGC
Eqca-DRB1*0601            GCGCTTCGACAGCGACGTGGGGGAGTTCCGGGCGCTGACCGAGCTGGGGC
Eqgr-DRB1*0101            GCGCTTCGACAGCGACGTGGGGGAGTTCCGGGCGGTGACCGAGCTGGGGC
Eqbu-DRB1*0101            GCGCTTCGACAGCGACGTGGGGGAGTTCCGGGCGGTGACCGAGCTGGGGC
Eqbu-DRB1*0201            GCGCTTCGACAGCGACGTGGGGGAGTTCCGGGCGGTGACTGAGCTGGGGC
Eqbu-DRB1*0301            GCGCTTCGACAGCGACGTGGGGGAGTTCCGGGCGGTGACTGAGCTGGGGC
Eqas-DRB1*0101            GCGCTTCGACAGCGACGTGGGGGAGTACCGGGCGGTGACCGAGCTGGGGC
Eqbu-DRB1*0401            GCGCTTCGACAGCGACGTGGGCGAGTACCGGGCGCTGACCGAGCTGGGGC
Eqas-DRB1*0201            GCGCTTCGACAGCGACGTGGGGGAGGTCCGGGCGGTGACCGAGCTGGGGC
Eqhe-DRB1*0101            GCGCTTCGACAGCGACGTGGGGGAGTTCCGGGCGGTGACCGAGCTGGGGC
Eqki-DRB1*0101            GCGCTTCGACAGCGACGTGGGGGAGTTCCGGGCGGTGACCGAGCTGGGAC
Eqki-DRB1*0201            GCGCTTCGACAGCGACGTGGGCGAGTACCGGGCGCTGACCGAGCTGGGGC
Eqca-DRB2*00101           GCGCTTCGACAGCGACGTGGGGGAGTACCGGGCGGTGACCGAGCTGGGGC
Eqca-DRB2*00201           GCGCTTCGACAGCGACGTGGGGGAGTACCGGGCGGTGACCGAGCTGGGGC
Eqca-DRB2*00301           GCGCTTCGACAGCGACGTGGGGGAGTACCGGGCGGTGACCGAGCTGGGGC
(Eqca-DRB2*00401)         GCGCTTCGACAGCGACGTGGGGGAGTACCGGGCGGTGACCGAGCTGGGGC
Eqca-DRB2*0501            GCGCTTCGACAGCGACGTGGGGGAGTACCGGGCGGTGACCAAGCTGGGGC
Eqca-DRB2*0601            GCGCTTCGACAGCGACGTGGGGGAGTACCGGGCGGTGACCGAGCTGGGGC
Eqca-DRB2*0701            GCGCTTCGACAGCGACGTGGGGGAGTACCGGGCGGTGACCAAGCTGGGGC
Eqpr-DRB2*0101            GCGCTTCGACAGCGACGTGGGGGAGTACCGGGCGGTGACCGAGCTGGGGC
Eqpr-DRB2*0201            GCGCTTCGACAGCGACGTGGGGGAGTTCCGGGCGGTGACCGAGCTGGGGC
Eqgr-DRB2*0101            GCGCTTCGACAGCGACGTGGGGGAGTACCGGGCGGTGACCGAGCTGGGGC
Eqze-DRB2*0101            GCGCTTCGACAGCGACGTGGGGGAGTACCGGGCGGTGACCGAGCTGGGGC
Eqbu-DRB2*0101            GCGCTTCGACAGCGACGTGGGGGAGTACCGGGCGGTGACCGAGCTGGGGC
Eqbu-DRB2*0201            GCGCTTCGACAGCGACGTGGGGGAGTACCGGGCGGTGACCGAGCTGGGGC
Eqbu-DRB2*0301            GCGCTTCGACAGCGACGTGGGGGAGTACCGGGCGCTGACCGAGCTGGGGC
Eqbu-DRB2*0401            GCGCTTCGACAGCGACGTGGGGGAGTACCGGGCGGTGACCGAGCTGGGGC
Eqbu-DRB2*0501            GCGCTTCGACAGCGACGTGGGGGAGTACCGGGCGGTGACCGAGCTGGGGC
Eqas-DRB2*0101            GCGCTTCGACAGCGACGTGGGGGAGTACCGGGCGGTGACCGAGCTGGGGC
Eqas-DRB2*0201            GCGCTTCGACAGCGACGTGGGGGAGTTCCGGGCGGTGACCGAGCTGGGGC
Eqas-DRB2*0301            GCGCTTCGACAGCGACGTGGGGGAGTACCGGGCGGTGACCGAGCTGGGGC
Eqhe-DRB2*0101            GCGCTTCGACAGCGACGTGGGCGAGTACCGGGCGGTGACCGAGCTGGGGC
Eqhe-DRB2*0201            GCGCTTCGACAGCGACGTGGGGGAGTACCGGGCGGTGACCGAGCTGGGGC
Eqki-DRB2*0101            GCGCTTCGACAGCGACGTGGGGGAGTTCCGGGCCGTGACCGAGCTGGGGC
Eqca-DRB3*00101           GCGCTTCGACAGCGACGTGGGCGAGTACCGGGCGCTGACCGAGCTGGGGC
Eqca-DRB3*00201           GCGCTTCGACAGCGACCTGGGCGAGTACCGGGCGCTGACCGAGCTGGGGC
Eqca-DRB3*0301            GCGCTTCGACAGCGACCTGGGCGAGTACCGGGCGCTGACCGAGCTGGGGC
Eqgr-DRB3*0101            GCGCTTCGACAGCGACGTGGGGGAGTACCGGGCGCTGACCGAGCTGGGGC
Eqze-DRB3*0101            GCGCTTCGACAGCGACGTGGGCGAGTACCGGGCGCTGACCGAGCTGGGGC
Eqbu-DRB3*0101            GCGCTTCGACAGCGACGTGGGCGAGTACCGGGCGCTGACCGAGCTGGGGC
Eqbu-DRB3*0201            GCGCTTCGACAGCGACGTGGGCGAGTACCGGGCGCTGACCGAGCTGGGGC
Eqbu-DRB3*0301            GCGCTTCGACAGCGACGTGGGCGAGTACCGGGCGCTGACCGAGCTGGGGC
Eqbu-DRB3*0401            GCGCTTCGACAGCGACGTGGGCGAGTACCGGGCGCTGACCGAGCTGGGGC
Eqas-DRB3*0101            GCGCTTCGACAGCGACGTGGGCGAGTACCGGGCGCTGACCGAGCTGGGGC
Eqas-DRB3*0201            GCGCTTCGACAGCGACGTGGGGGAGTTCCGGGCGCTGACCGAGCTGGGGC
Eqas-DRB3*0301            GCGCTTCGACAGCGACGTGGGCGAGTACCGGGCGCTGACCGAGCTGGGGC
Eqhe-DRB3*0101            GCGCTTCGACAGCGACGTGGGCGAGTACCGGGCGCTGACCGAGCTGGGGC
Eqhe-DRB3*0201            GCGCTTCGACAGCGACGTGGGCGAGTACCGGGCGCTGAACGAGCTGGGGC
Eqhe-DRB3*0301            GCGCTTCGACAGCGACGTGGGCGAGTACCGGGCGCTGACCGAGCTGGGGC
Eqki-DRB3*0101            GCGCTTCGACAGCGACGTGGGCGAGTACCGGGCGCTGACCGAGCTGGGGC
```

```
                          160       170       180       190       200
                 ....|....|....|....|....|....|....|....|....|....|
Eqca-DRB1*00101  GGCCGGACGCCGAGTACTGGAACGGGCAGCAGGACATCCTGGATGAACGG
(Eqca-DRB1*00201) GGCCGGACGCCGAGAACTGGAACGGGCAGAAGGACTTCCTGGATGACCGG
(Eqca-DRB1*00301) GGCCGGACGCCGAGTACTGGAACGGGCAGAAGGACGTCCTGGATGACGCG
(Eqca-DRB1*00401) GGCCGGACGCCGAGTACTGGAACGGGCAGAAGGACTTCCTGGATGACGCG
(Eqca-DRB1*00501) GGCCGGACGCCGAGTACTGGAACGGGCAGAAGGACTTCCTGGAGCGGAAG
Eqca-DRB1*0601   GGCCTGGCGCCGAGTTCCGGAACCGGGAGAAGGACTTCCTGGAGCAGAAT
Eqgr-DRB1*0101   GGCCGGACGCCGAGTACCTGAACAGGCAGAAGGACTTCCTGGAGCAGACG
Eqbu-DRB1*0101   GGCGGGACGCCGAGTACCTGAATGGGCAGAAGGACTTCCTGGAGCAGAAG
Eqbu-DRB1*0201   GGCCGACCGCCGAGAAGTGGAACGGGCAGAAGGACTTCCTGGAGCGGGCG
Eqbu-DRB1*0301   GGCCGACCGCCGAGTACTGGAACAGACAGAAGGACTTCCTGGATGACGCG
Eqas-DRB1*0101   GGCCGATCGCCGAGTACCTGAACCGGCAGAAGGACTTCCTGGATGACGCG
Eqbu-DRB1*0401   GGCGGAGCGCCGAGTACTGGAACGGGCAGCAGGACATCCTGGAGCAGAAG
Eqas-DRB1*0201   GGCCGGACGCCGAGTACCTGAATGGGCAGAAGGACTTCCTGGAGCAGCAG
Eqhe-DRB1*0101   GGCCGGACGCCGAGTACCTGAATGGGCAGAAGGACTTCCTGGAGCAGAAG
Eqki-DRB1*0101   GGCGGGACCCCGAGTACCTGAATGGGCAGAAGGACTTCCTGGAGCAGCAG
Eqki-DRB1*0201   GGCGGAGCGCCGAGTACTGGAACGGGCAGAAGGACGTCCTGGACCAGAAG
Eqca-DRB2*00101  GGCCGGACGCCGAGTACTGGAACGGGCAGAAGGACGTCCTGGATGACGCG
Eqca-DRB2*00201  GGCGGAGCGCCGAGTACTGGAACGGGCAGAAGGACGTCCTGGATGACGCG
Eqca-DRB2*00301  GGCCGGACGCCGAGTACTGGAACGGGCAGAAGGACGTCCTGGATGACGCG
(Eqca-DRB2*00401) GGCCGGACGCCGAGTACTGGAACGGGCAGAAGGACGTCCTGGATGACGCG
Eqca-DRB2*0501   GGACGGACGCCGAGTACTAGAACGGGCAGAAGGACGTCCTGGATGACGCG
Eqca-DRB2*0601   GGCGGAGCGCCGAGTACTGGAACGGGCAGAAGGACGTCCTGGATGACGCG
Eqca-DRB2*0701   GGACGGACGCCGAGTACTGGAACGGGCAGAAGGACGTCCTGGATGACGCG
Eqpr-DRB2*0101   GGCGGAGCGCCGAGTACTGGAACGGGCAGAAGGACGTCCTGGATGACGCG
Eqpr-DRB2*0201   GGCGGAGCGCCGAGTACTAGAACGGGCAGAAGGACGTCCTGGATGACGCG
Eqgr-DRB2*0101   GGCCGGACGCCGAGTACTGGAACGGGCAGAAGGACGTCCTGGATGACGCG
Eqze-DRB2*0101   GGCGGAGCGCCGAGTACTGGAACGGGCAGAAGGACGTCCTGGATGACGCG
Eqbu-DRB2*0101   GGCGGAGCGCCGAGTACTGGAACGGGCAGAAGGACGTCCTGGATGACGCG
Eqbu-DRB2*0201   GGCGGAGCGCCGAGTACTGGAACGGGCAGAAGGACGTCCTGGATGACGCG
Eqbu-DRB2*0301   GGCGGAGCGCCGAGTACTGGAACGGGCAGAAGGACGTCCTGGATGACGCG
Eqbu-DRB2*0401   GGCCGGACGCCGAGTACTGGAACGGGCAGAAGGACGTCCTGGATGACGCG
Eqbu-DRB2*0501   GGCCGGACGCCGAGTACTGGAACGGGCAGAAGGACGTCCTGGATGACGCG
Eqas-DRB2*0101   GGCGGAGCGCCGAGTACTGGAACGGGCGGAAGGACATCCTGGAGCGGAAG
Eqas-DRB2*0201   GGCCGGACGCCGAGTACTGGAACGGACAGAAGGACGTCCTGGAGCGGAAG
Eqas-DRB2*0301   GGCGGAGCGCCGAGTACCTGAACGGGCAGAAGGACTTCCTGGATGACGCG
Eqhe-DRB2*0101   GGCGGAGCGCCGAGTACTGGAACGGGCGGAAGGACTTCCTGGAGCAGAAG
Eqhe-DRB2*0201   GGCCGGACGCCGAGTACTGGAACGGGCGGAAGGACATCCTGGAGCAGAAG
Eqki-DRB2*0101   GGCGGAGCGCCGAGTACTGGAACGGGCGGAAGGACATCCTGGAGCAGAAG
Eqca-DRB3*00101  GGCGGAGCGCCGAGTACTGGAACGGGCAGCAGGACATCCTGGAGCAGAAG
Eqca-DRB3*00201  GGCCGGACGCCGAGTACTGGAACGGGCAGCAGGACATCCTGGAGCAGAAG
Eqca-DRB3*0301   GGCCGGACGCCGAGTACTGGAACGGGCAGCAGGACATCCTGGAGCAGAAG
Eqgr-DRB3*0101   GGCCGGACGCCGAATACTGGAACGGGCAGAAGGACATCCTGGAGCAGAAG
Eqze-DRB3*0101   GGCGGAGCGCCGAGTACTGGAACGGGCAGCAGGACATCCTGGAGCAGAAG
Eqbu-DRB3*0101   GGCGGAGCGCCGAGTACTGGAACGGGCAGCAGGACATCCTGGAGCAGAAG
Eqbu-DRB3*0201   GGCCGGACGCCGAGTACTGGAACGGGCAGCAGGACATCCTGGAGCAGAAG
Eqbu-DRB3*0301   GGCGGAGCGCCGAGTACTGGAACGGACAGAAGGACATCCTGGAGCAGAAG
Eqbu-DRB3*0401   GGCGGAGCGCCGAGTACTGGAACGGGCAGCAGGACATCCTGGAGCAGAAG
Eqas-DRB3*0101   GGCGGAGCGCCGAGTACCTGAACGGGCAGAAGGACTTCCTGGAGCAGAAG
Eqas-DRB3*0201   GGCGGAGCGCCGAGTACTGGAACGGGCAGAAGGACTTCCTGGAGCAGAAG
Eqas-DRB3*0301   GGCGGAGCGCCGAGTACTGGAACGGGCAGAAGGACTTCCTGGAGCAGAAG
Eqhe-DRB3*0101   GGCGCAGCGCCGAGTACTGGAACGGACAGAAGGACTTCCTGGAGCAGAAG
Eqhe-DRB3*0201   GGCGGAGCGCCGAGTACTGGAACGGGCAGAAGGACTTCCTGGAGCAGAAG
Eqhe-DRB3*0301   GGCGGGACGCCGAGTACTGGAACGGGCAGAAGGACGTCCTGGACCAGAAG
Eqki-DRB3*0101   GGCGGAGCGCCGAGTACTGGAACGGGCAGAAGGACGTCCTGGACCAGAAG
```

```
                       210       220       230       240       250
              ....|....|....|....|....|....|....|....|....|....|
Eqca-DRB1*00101    CGGGCAGCGGTGGACACGTACTGCAGACACAACTACGGGGTTATTGATGG
(Eqca-DRB1*00201)  CGGGCCTCGGTGGACACGTACTGCAGACACAACTACGGCGTCCTTGACAA
(Eqca-DRB1*00301)  CGGGCCCAGGTGGACACGTACTGCAGACACAACTACGGCATCAGCGACAG
(Eqca-DRB1*00401)  CGGGCCGCGGTGGACACGTTGTGCAGGCACAACTACGGCATCAGCGAGAG
(Eqca-DRB1*00501)  CGGGCCGAGGTGGACACGTTCTGCAGACACAACTACGGCGTCAGCGAGAG
Eqca-DRB1*0601     CGGGCCGCAGTGGACACGTACTGCAGACACAACTACGGCGTCATCGAGAA
Eqgr-DRB1*0101     CGGGCCGCGGTGGACACCGTGTGCAGACACAACTACGGCGTCAGCGAGGG
Eqbu-DRB1*0101     CGGGCCGCGGTGGACACGTACTGCAGGCACAACTACGGTGTCAGCGAGGG
Eqbu-DRB1*0201     CGGGCCGCGGTGGACACGTACTGCAGACACAACTACGGCATCTTTGACAA
Eqbu-DRB1*0301     CGGGCCGCGGTGGACACGGTGTGCAGGCACAACTACGGCGTCAGCGAGAA
Eqas-DRB1*0101     CGGGCCGCGGTGGACACGTACTGCAGACACAACTACGGCGTCCTTGACAA
Eqbu-DRB1*0401     CGGGCGGAGGTGGACACGGTGTGCAGACACAACTACGGCGTCAGCGAGAG
Eqas-DRB1*0201     CGGGCCGCGGTGGACAAGTACTGCAGGCACAACTACGGTGTCAGCGAGGG
Eqhe-DRB1*0101     CGGGCCGCGGTGGACACGGTGTGCAGACACAACTACGGCGTCAGCGAGAG
Eqki-DRB1*0101     CGGGCCGCGGTGGACACGGTGTGCAGGCACAACTACGGTGTCAGCGAGGG
Eqki-DRB1*0201     CGGGCGGAGGTGGACACGGTGTGCAGACACAACTACGGCGTCAGCGAGAG
Eqca-DRB2*00101    CGGGCCGCGGTGGACACGTACTGCAGACACAACTACGGCGTCAGCGACAG
Eqca-DRB2*00201    CGGGCCGCGGTGGACACGTACTGCAGACACAACTACGGCGTCAGCGACAG
Eqca-DRB2*00301    CGGGCCGCGGTGGACACGTACTGCAGACACAACTACAGGGTTTTTGATAG
(Eqca-DRB2*00401)  CGGGCCGCGGTGGACACGTACTGCAGACACAACTACGCCGTCAGCGAGAG
Eqca-DRB2*0501     CGGGCCGCGGTGGACACGTACTGCAGACACAACTACGGCGTCAGCGACAG
Eqca-DRB2*0601     CGGGCCGCGGTGGACACGTACTGCAGACACAACTACAGGGTTTTTGATAG
Eqca-DRB2*0701     CGGGCCGCGGTGGACACGTACTGCAGACACAACTACGGCGTCAGCGACAG
Eqpr-DRB2*0101     CGGGCCGCGGTGGACACGTACTGCAGACACAACTACGGCGTCAGCGACAG
Eqpr-DRB2*0201     CGGGCCGCGGTGGACACGTACTGCAGACACAACTACGGCGTCAGCGACAG
Eqgr-DRB2*0101     CGGGCCGCGGTGGACACGTACTGCAGACACAACTACAGGGTTTTTGATAG
Eqze-DRB2*0101     CGGGCCGCGGTGGACACGTACTGCAGACACAACTACGGCGTCAGCGAGAG
Eqbu-DRB2*0101     CGGGCCGCGGTGGACACGTACTGCAGACACAACTACGGCGTCAGCGAGAG
Eqbu-DRB2*0201     CGGGCCGCGGTGGACACGTACTGCAGACACAACTACGGCGTCAGCGAGAG
Eqbu-DRB2*0301     CGGGCCGCGGTGGACACGTACTGCAGACACAACTACGGCGTCAGCGAGAG
Eqbu-DRB2*0401     CGGGCCGCGGTGGACACGTACTGCAGACACAACTACGGCGTCAGCGAGAG
Eqbu-DRB2*0501     CGGGCCGCGGTGGACACGTACTGCAGACACAACTACGGCGTCAGCGAGAG
Eqas-DRB2*0101     CGGGCCGCGGTGGACACGTACTGCAGACACAACTACGGCGTCAGCGAGAG
Eqas-DRB2*0201     CGGGCCGCGGTGGACGCGTACTGCAGACACAACTACGGCGTCCTTGACAA
Eqas-DRB2*0301     CGGGCCGCGGTGGACACGTACTGCAGACACAACTACGGCGTCAGCGAGAG
Eqhe-DRB2*0101     CGGGCCGCGGTGGACACGTACTGCAGACACAACTACGGCGTCAGCGAGAG
Eqhe-DRB2*0201     CGGGCCGCGGTGGACACGTACTGCAGACACAACTACGGCGTCAGCGAGAG
Eqki-DRB2*0101     CGGGCCGCGGTGGACACGTACTGCAGACACAACTACGGCGTCAGCGAGAG
Eqca-DRB3*00101    CGGGCCAAGGTGGACACGTACTGCAGACACAACTACGCCGTCAGCGAGAG
Eqca-DRB3*00201    CGGGCGGAGGTGGACACGGTGTGCAGACACAACTACGGCGTCAGCGAGAG
Eqca-DRB3*0301     CGGGCGGAGGTGGACACGGTGTGCAGACACAACTACGGCGTCAGCGAGAG
Eqgr-DRB3*0101     CGGGCGGAGGTGGACACGGTGTGCAGACACAACTACGGCGTCAGCGAGAG
Eqze-DRB3*0101     CGGGCGGAGGTGGACACGGTGTGCAGACACAACTACGGCGTCAGCGAGAG
Eqbu-DRB3*0101     CGGGCGGAGGTGGACACGGTGTGCAGACACAACTACGGCGTCAGCGAGAG
Eqbu-DRB3*0201     CGGGCGGAGGTGGACACGGTGTGCAGACACAACTACGGCGTCAGCGAGAG
Eqbu-DRB3*0301     CGGGCGGAGGTGGACACGGTGTGCAGACACAACTACGGCGTCAGCGAGAG
Eqbu-DRB3*0401     CGGGCGGAGGTGGACACGGTGTGCAGACACAACTACGGCGTCAGCGAGAG
Eqas-DRB3*0101     CGGACGGAGGTGGACACGGTGTGCAGACACAACTACGGCGTCAGCGAGAG
Eqas-DRB3*0201     CGGGCGGAGGTGGACACGGTGTGCAGACACAACTACGGCGTCAGCGAGAG
Eqas-DRB3*0301     CGGGCGGAGGTGGACACGGTGTGCAGACACAACTACGGCGTCAGCGAGAG
Eqhe-DRB3*0101     CGGGCGGAGGTGGACACGGTGTGCAGACACAACTACGGCGTCAGCGAGAG
Eqhe-DRB3*0201     CGGGCCGAGCTGGACACGGTGTGCAGACACAACTACGGCGTCAGCGAGAG
Eqhe-DRB3*0301     CGGGCGGAGGTGGACACGGTGTGCAGACACAACTACGGCGTCAGCGAGAG
Eqki-DRB3*0101     CGGGCGGAGGTGGACACGGTGTGCAGACACAACTACGGCGTCAGCGAGAG
```

```
                          260
                 ....|....|
Eqca-DRB1*00101  CTTCCTGGTG
(Eqca-DRB1*00201) CTTCCTGGTG
(Eqca-DRB1*00301) CTTCCTGGTG
(Eqca-DRB1*00401) CTTCCTGGTG
(Eqca-DRB1*00501) CTTCCTGGTG
Eqca-DRB1*0601   CTTCCTGGTG
Eqgr-DRB1*0101   CTTCCTGGTG
Eqbu-DRB1*0101   CTTCCTGGTG
Eqbu-DRB1*0201   CTTCCTGGTG
Eqbu-DRB1*0301   CTTCCTGGTG
Eqas-DRB1*0101   CTTCCTGGTG
Eqbu-DRB1*0401   CTTCCTGGTG
Eqas-DRB1*0201   CTTCCTGGTG
Eqhe-DRB1*0101   CTTCCTGGTG
Eqki-DRB1*0101   CTTCCTGGTG
Eqki-DRB1*0201   CTTCCTGGTG
Eqca-DRB2*00101  CTTCCTGGTG
Eqca-DRB2*00201  CTTCCTGGTG
Eqca-DRB2*00301  CCTCCTGGTG
(Eqca-DRB2*00401) CTTCCTGGTG
Eqca-DRB2*0501   CTTCCTGGTG
Eqca-DRB2*0601   CCTCCTGGTG
Eqca-DRB2*0701   CTTCCTGGTG
Eqpr-DRB2*0101   CTTCCTGGTG
Eqpr-DRB2*0201   CTTCCTGGTG
Eqgr-DRB2*0101   CCTCCTGGTG
Eqze-DRB2*0101   CTTCCTGGTG
Eqbu-DRB2*0101   CTTCCTGGTG
Eqbu-DRB2*0201   CTTCCTGGTG
Eqbu-DRB2*0301   CTTCCTGGTG
Eqbu-DRB2*0401   CTTCCTGGTG
Eqbu-DRB2*0501   CTTCCTGGTG
Eqas-DRB2*0101   CTTCCTGGTG
Eqas-DRB2*0201   CTTCCTGGTG
Eqas-DRB2*0301   CTTCCTGGTG
Eqhe-DRB2*0101   CTTCCTGGTG
Eqhe-DRB2*0201   CTTCCTGGTG
Eqki-DRB2*0101   CTTCCTGGTG
Eqca-DRB3*00101  CTTCCTGGTG
Eqca-DRB3*00201  CTTCCTGGTG
Eqca-DRB3*0301   CTTCCTGGTG
Eqgr-DRB3*0101   CTTCCTGGTG
Eqze-DRB3*0101   CTTCCTGGTG
Eqbu-DRB3*0101   CTTCCTGGTG
Eqbu-DRB3*0201   CTTCCTGGTG
Eqbu-DRB3*0301   CTTCCTGGTG
Eqbu-DRB3*0401   CTTCCTGGTG
Eqas-DRB3*0101   CTTCCTGGTG
Eqas-DRB3*0201   CTTCCTGGTG
Eqas-DRB3*0301   CTTCCTGGTG
Eqhe-DRB3*0101   CTTCCTGGTG
Eqhe-DRB3*0201   CTTCCTGGTG
Eqhe-DRB3*0301   CTTCCTGGTG
Eqki-DRB3*0101   CTTCCTGGTG
```

10 20 30 40 50

```
                      ....|....|....|....|....|....|....|....|....|....|
Eqca-DQB1*00101       AGGATTTCGTGATCCAGCTTAAGGGCCTGTGCTACTTCACCAACGGGACG
(Eqca-DQB1*00201)     AGGATTTCCTGCTCCAGTTTAAAGGCCTGTGCTACTTCACCAACGGGACG
(Eqca-DQB1*00301)     AGGATTTCGTGCACCAGTTTAAAGGCCTGTGCTACTTCACCAACGGGACG
(Eqca-DQB1*00401)     AGGATTTCGTGTACCAGCTTAAGGGCCTGTGCTACTTCACCAACGGGACG
Eqca-DQB1*00501       AGGATTTCGTGTACCAGCTTAAGTTTCTGTGCTACTTCACCAACGGGACG
Eqca-DQB1*0601        AGGATTTCCTGTTCCAGTTTAAGGGCCTTTGCTACTTCACCAACGGGACG
Eqca-DQB1*0701        AGGATTTCGTGATCCAGCTTAAGGGCCTGTGCTACTTCACCAACGGGACG
Eqca-DQB1*0801        AGGATTTCCTGTTCCAGTTTAAGGGCCTTTGCTACTTCACCAACGGGACG
Eqpr-DQB1*0101        AGGATTTCGTGTACCAGCTTAAGTTTCTGTGCTACTTCACCAACGGGACG
Eqgr-DQB1*0101        AGGATTTCGTGTACCAGCTTAAGTTTCTGTGCTACTTCACCAACGGGACG
Eqze-DQB1*0101        AGGATTTCGTGTACCAGCTTAAGTTTCTGTGCTACTTCACCAACGGGACG
Eqbu-DQB1*0101        AGGATTTCGTGTACCAGCTTAAGTTTCTGTGCTACTTCACCAACGGGACG
Eqbu-DQB1*0201        AGGATTTCGTGTACCAGCTTAAGGCCCTGTGCTACTTCACCAACGGGACG
Eqbu-DQB1*0301        AGGATTTCCTGTTCCAGTTTAAAGGCCAGTGCTACTACACCAACGGGACG
Eqbu-DQB1*0401        AGGATTTCCTGTTCCAGTTTAAAGGCCAGTGCTACTACACCAACGGGACG
Eqbu-DQB1*0501        AGGATTTCCTGTTCCAGTTTAAGGGCCTGTGCTACTTCACCAACGGGACA
Eqas-DQB1*0101        AGAATTTCCTGTTCCAGTTTAAGGGCCTGTGCTACTTCACCAACGGGACG
Eqhe-DQB1*0101        AGGATTTCGTGTACCAGCTTAAGTTTCTGTGCTACTTCACCAACGGGACG
Eqhe-DQB1*0201        AGGATTTCCTGTACCAGTTTAAAGGCCTGTGCTACTTCACCAACGGGACG
Eqki-DQB1*0101        AGGATTTCGTGCACCAGTTTAAGGGCCTGTGCTACTTCACCAACGGGACG
Eqki-DQB1*0201        AGGATTTCCTGTTCCAGTTTAAGGGCCTGTGCTACTTCACCAACGGGACG
Eqca-DQB2*00101       AGGATTTCGTGGTCCAGTTTATGGGCCAGTGCTACTTCACCAACGGGACG
(Eqca-DQB2*00201)     AGGATTTCGTGTACCAGTTTAAGGGCCAGTGCTACTTCACCAACGGGACG
(Eqca-DQB2*00301)     AGGATTTCGTGATCCAGTTTAAGGCCCAGTGCTACTTCACCAATGGGACG
Eqca-DQB2*00401       CGGATTTCGTGTACCAGTTTATGGGCCAGTGCTACTTCACTAACGGGACG
(Eqca-DQB2*00501)     AGGATTTCGTGTACCAGTTTAAGTTTCATTGCTATTTCATCAACGGAACG
Eqca-DQB2*0601        AGGATTTCGTGTTCCAGGCTGTTGGCCAGTGCTACTTCACCAACGGGACG
Eqca-DQB2*0701        CGGATTTCGTGCACCAGTTTATGGGCCAGTGCTACTTCACCAACGGGACG
Eqca-DQB2*0801        CGGATTTCGTGTACCAGTTTATGGGCCAGTGCTACTTCACTAACGGGACG
Eqpr-DQB2*0101        CGGATTTCGTGTACCAGTTTATGGGCCAGTGCTACTTCACTAACGGGACG
Eqpr-DQB2*0201        AGGATTTCGTGTACCTGTTTATGGGCCAGTGCTACTTCACCAACGGGACG
Eqgr-DQB2*0101        AGGATTTCGTGTACCAGTTTATGGGCCAGTGCTACTTCACCAACGGGACG
Eqze-DQB2*0101        AGGATTTCGTGTACCAGTTTATGGGCCAGTGCTACTTCACCAACGGGACG
Eqbu-DQB2*0101        AGGATTTCGTGGTCCAGTTTATGGGCCAGTGCTACTTCACCAACGGGACG
Eqbu-DQB2*0201        TGGATTTCGTGTACCAGTTTATGGGCCAGTGCTACTTCACCAACGGGACG
Eqbu-DQB2*0301        AGGATTTCGTGTACCAGTTTATGGGCCAGTGCTACTTCACCAACGGGACG
Eqbu-DQB2*0401        TGGATTTCGTGTACCAGTTTATGGGCCAGTGCTACTTCACCAACGGGACG
Eqas-DQB2*0101        TGGATTTCGTGTACCAGTTTATGGGCCAGTGCTACTTCACCAACGGGACG
Eqas-DQB2*0201        TGGATTTCGTGTACCAGTTTATGGGCCAGTGCTACTTCACCAACGGGACG
Eqas-DQB2*0301        TGGATTTCGTGTACCAGTTTATGGGCCAGTGCTACTTCACCAACGGGACG
Eqki-DQB2*0101        AGGATTTCGTGGTCCAGTTGATGGGCCAGTGCTACTTCACCAACGGGACG
Eqca-DQB3*00101       AGGATTTCGTGTTCCAGCTTAAGGGCCAGTGCTACTTCACCAACGGGACG
(Eqca-DQB3*00201)     --------------CAGCTTAAGGGCCAGTGCTACTTCACCAACGGGACG
Eqca-DQB3*0301        AGGATTTCGTGTTCCAGCTTAAGGGCCAGTGCTACTTCACCAACGGGACG
Eqca-DQB3*0401        AGGATTTCGTGTTCCAGCTTAAGGGCCAGTGCTACTTCACCAACGGGACG
Eqca-DQB3*0501        AGGATTTCGTGTTCCAGCTTAAGGGCCAGTGCTACTTCA---ACGGGACG
Eqpr-DQB3*0101        AGGATTTCGTGTTCCAGCTTAAGGGCCAGTGCTACTTCACCAACGGGACG
Eqpr-DQB3*0201        AGGATTTCGTGTTCCAGCTTAAGGGCCAGTGCTACTTCACCAACGGGACG
Eqpr-DQB3*0301        AGGATTTCGTGTTCCAGCTTAAGGGCCAGTGCTACTTCA---ACGGGACG
Eqpr-DQB3*0401        AGGATTTCGTGTTCCAGCTTAAGGGCCAGTGCTACTTCA---ACGGGACG
Eqgr-DQB3*0101        AGGATTTCGTGTTCCAGCTTAAGGGCCAGTGCTACTTCACCAACGGGACG
Eqgr-DQB3*0201        AGGATTTCGTGTTCCAGCTTAAGGGCCAGCGCTACTTCACCAACGGGACG
Eqze-DQB3*0101        AGGATTTCGTGTTCCAGCTTAAGGGCCAGTGCTACTTCACCAACGGGACG
Eqbu-DQB3*0101        AGGATTTCGTGTTCCAGCTTAAGGGCCAGTGCTACTTCACCAACGGGACG
Eqbu-DQB3*0201        AGGATTTCGTGTTCCAGCTTAAGGGCCAGTGCTACTTCACCAACGGGACG
Eqbu-DQB3*0301        AGGATTTCGTGTTCCAGCTTAAGGGCCAGTGCTACTTCACCAACGGGACG
Eqbu-DQB3*0401        AGGATTTCGTGTTCCAGCTTAAGGGCCAGTGCTACTTCACCAACGGGACG
Eqas-DQB3*0101        AGGATTTCGTGTTCCAGCTTAAGGGCCAGTGCTACTTCACCAACGGGACG
Eqas-DQB3*0201        AGGATTTCGTGTTCCAGCTTAAGGGCCAGTGCTACTTCACCAACGGGACG
Eqas-DQB3*0301        AGGATTTCGTGTTCCAGCTTAAGGGCCAGTGCTACTTCACCAACGGGACG
                               10        20        30        40        50
```

```
                   ....|....|....|....|....|....|....|....|....|....|
Eqhe-DQB3*0101     AGGATTTCGTGTTCCAGCTTAAGGGCCAGTGCTACTTCACCAACGGGACG
Eqhe-DQB3*0201     AGGATTTCGTGTTCCAGCTTAAGGGCCAGTGCTACTTCACCAACGGGACG
Eqki-DQB3*0101     AGGATTTCGTGTTCCAGCTTAAGGGCCAGTGCTACTTCACCAACGGGACG
Eqki-DQB3*0201     AGGATTTCGTGTTCCAGCTTAAGGGCCAGTGCTACTTCACCAACGGGACG
Eqki-DQB3*0301     AGGATTTCGTGTTCCAGCTTAAGGGCCAGTGCTACTTCACCAACGGGACG
Eqca-DQBPr*0101    AGGATTTCGTGATCCAGTTTAAGGCCCAGTGCTACTTCACCAATGGGACG
Eqze-DQBPr*0101    AGGATTTCGTGATCCAGTTTAAGGGCCAGTGCTACTTCACCAATGGGACC
Eqbu-DQBPr*0101    AGGATTTCGTGATCCAGTTTAAGGGCCAGTGCTACTTCACCAATGGGACC
Eqbu-DQBPr*0201    AGGATTTCGTGATCCAGTTTAAGGCCCAGTGCTACTTCACCAATGGGACG
Eqbu-DQBPr*0301    AGGATTTCGTGATCCAGTTTAAGGCCCAGTGCTACTTCACCAATGGGACG
Eqbu-DQBPr*0401    AGGATTTCGTGATCCAGTTTAAGGCCCAGTGCTACTTCACCAATGGGACG
Eqas-DQBPr*0101    AGGATTTCGTGCACCAGTTTAAGGGCCAGTGCTACTTCACCAACGGGACG
Eqas-DQBPr*0201    AGGATTTCGTGATCCAGTTTAAGGCCCAGTGCTACTTCACCAATGGGACG
Eqhe-DQBPr*0101    AGGATTTCGTGATCCAGTTTAAGGGCCAGTGCTACTTCACCAATGGGACG
Eqhe-DQBPr*0201    AGGATTTCGTGCACCAGTTTAAGGGCCAGTGCTACTTCACCAACGGGACG
Eqki-DQBPr*0101    AGGATTTCGTGATCCAGTTTAAGGGCCAGTGCTACTTCACCAATGGGACC

                             60        70        80        90       100
                   ....|....|....|....|....|....|....|....|....|....|
Eqca-DQB1*00101    GAGCGGGTGCGGCTCGTGACCAGATACATCTACAACCGGGAGGAGTGGGT
(Eqca-DQB1*00201)  GAGCGGGTGCGGAGTGTGAACAGATACATCTACAACCGGGAGGAGTACGT
(Eqca-DQB1*00301)  GAGCGGGTGCGGCACGTGACCAGATACATCTACAACCGGGAGGAGTTCGT
(Eqca-DQB1*00401)  GAGCGGGTCCGGCTCGTGGCCAGATGGCTCTACAACCGGGAGGAGTTCGT
Eqca-DQB1*00501    GAGCGGGTGCGGCTCTTGGCGAGATACCTCTACAACCGGGAGGAGTGGGT
Eqca-DQB1*0601     GAGCGCGTGCGGTTCGTGACCAGATACCTCTACAACCGGGAGGAATTCCT
Eqca-DQB1*0701     GAGCGGGTGCGGCACGTGACCAGATACATCTACAACCGGGAGGAGTGGGT
Eqca-DQB1*0801     GAGCGCGTGCGGTTCGTGACCAGATACCTCTACAACCGGGAGGAATTCCT
Eqpr-DQB1*0101     GAGCGGGTGCGGCTCTTGGCGAGATACCTCTACAACCGGGAGGAGTGGGT
Eqgr-DQB1*0101     GAGCGGGTGCGGCTCTTGGCGAGATACCTCTACAACCGGGAGGAGTGGGT
Eqze-DQB1*0101     GAGCGGGTGCGGCTCTTGGCGAGATACCTCTACAACCGGGAGGAGTGGGT
Eqbu-DQB1*0101     GAGCGGGTGCGGCTCTTGGCGAGATACCTCTACAACCGGGAGGAGTGGGT
Eqbu-DQB1*0201     GAGCGGGTGCGGCTCTTGGCGAGATACCTCTACAACCGGGAGGAGTGGGT
Eqbu-DQB1*0301     GAGCGGGTGCGGCTCGTGACCACATACATCTACAACCGGGAGGAGTGGGT
Eqbu-DQB1*0401     GAGCGGGTGCGGCTCGTGACCAGACTCATCTACAACCGGGAGGAGTGGGT
Eqbu-DQB1*0501     GAGCGCGTGCGGCTCGTGACCAGATACCTCTACAACCGGGAGGAGTTCGT
Eqas-DQB1*0101     GAGCGGGTGAGGCTCGTGACCAGATACCTCTACAACCGGGAGGAGCACCT
Eqhe-DQB1*0101     GAGCGGGTGCGGCTCTTGGCGAGATACCTCTACAACCGGGAGGAGTGGGT
Eqhe-DQB1*0201     GAGCGGGTGCGGCTCGTGACCAGATACCTCTACAACCGGGAGGAGTGGGT
Eqki-DQB1*0101     GAGCGCGTGCGGCTCGTGACCAGATACCTCTACAACCGGGAGGAGTTCGT
Eqki-DQB1*0201     GAGCGCGTGCGGCTCGTGACCAGATACCTCTACAACCGGGAGGAGTTCGT
Eqca-DQB2*00101    GAGCACGTGCGGTACGTGACCAGATACATCTACAACCGGGAGGAGTACGT
(Eqca-DQB2*00201)  GAGCGGGTGCGGCTCGTGACCAGATACATCTACAACCAGGAGGAGTACGC
(Eqca-DQB2*00301)  GAGCGGGTGCGGCTCGTGACCAGACTCATCTATAACCTGGAGGAGTACGC
Eqca-DQB2*00401    GAGCACGTGCGGTACGTGACCAGATACATCTACAACCGGGAGGAGTACGT
(Eqca-DQB2*00501)  GAGCGGGTGCGGAGTATGGAGAGATACATTTACAACCGGGAGGAGTTCGT
Eqca-DQB2*0601     CAGCACGTGCGGTACGTGACCAGATACATCTACAACCGGGAGGAGTTCGT
Eqca-DQB2*0701     GAGCACGTGCGGTACGTGACCAGATACATCTACAACCGGGAGGAGAACGT
Eqca-DQB2*0801     GAGCACGTGCGGTACGTGACCAGATACATCTACAACCGGGAGGAGTTCGT
Eqpr-DQB2*0101     GAGCACGTGCGGTACGTGACCAGATACATCTACAACCGGGAGGAGTTCGT
Eqpr-DQB2*0201     GAGCACGTGCGGTACGTGACCAGATTCGTCTACAACCGGGAGGAGTTCGT
Eqgr-DQB2*0101     CAGCACGTGCGGTACGTGACCAGATACATCTACAACCGGGAGGAGTACGT
Eqze-DQB2*0101     CAGCACGTGCGGTACGTGACCAGATACATCTACAACCGGGAGGAGTACGT
Eqbu-DQB2*0101     GAGCACGTGCGGTACTTGGTCAGTTACATCTACAACCGGGAGGAGTACGT
Eqbu-DQB2*0201     GAGCACGTGCGGTACGTGACCAGATACATCTACAACCGGGAGGAGTACGT
Eqbu-DQB2*0301     CAGCACGTGCGGTACGTGACCAGATTCATCTACAACCGGGAGGAGTTCGT
Eqbu-DQB2*0401     GAGCACGTGCGGTACGTGACCAGAATCATCTACAACCGGGAGGAGTTCGT
Eqas-DQB2*0101     CAGCACGTGCGGTACGTGACCAGATTCATCTACAACCGGGAGGAGTACGT
Eqas-DQB2*0201     GAGCACGTGCGGTACGTGACCAGATACATCTACAACCGGGAGGAGTACGT
Eqas-DQB2*0301     GAGCACGTGCGGTACGTGACCAGATACATCTACAACCGGGAGGAGAACGT
Eqki-DQB2*0101     CAGCACGTGCGGTACGTGACCAGATTCATCTACAACCGGGAGGAGTACGT
                             60        70        80        90       100
```

```
                     ....|....|....|....|....|....|....|....|....|....|
Eqca-DQB3*00101      GAGCGGGTGCGGCTCGTGACCAGACTCATCTACAACCGGGAGGAGTTCGT
(Eqca-DQB3*00201)    GAGCGGGTGCGGCTCGTGATCAGACTCATCTACAACCGGGAGGAGTTCGT
Eqca-DQB3*0301       GAGCGGGTGCGGCTCGTGACCAGACTCATCTACAACCGGGAGGAGTTCGT
Eqca-DQB3*0401       GAGCGGGTGCGGCTTGTGACCAGACTCATCTACAACCGGGAGGAGTTCGT
Eqca-DQB3*0501       GAGCGGGTGCGGCTTGTGACCAGACTCATCTACAACCGGGAGGAGTTCGT
Eqpr-DQB3*0101       GAGCGGGTGCGGCTCGTGACCAGACTCATCTACAACCGGGAGGAGTTCGT
Eqpr-DQB3*0201       GAGCGGGTGCGGCTCGTGACCAGACTCATCTACAACCGGGAGGAGTTCGT
Eqpr-DQB3*0301       GAGCGGGTGCGGCTTGTGACCAGACTCATCTACAACCGGGAGGAGTTCGT
Eqpr-DQB3*0401       GAGCGGGTGCGGCTTGTGACCAGACTCATCTACAACCGGGAGGAGTTCGT
Eqgr-DQB3*0101       GAGCGGGTGCGGCTCGTGACCAGACTCATCTACAACCGGGAGGAGTTCGT
Eqgr-DQB3*0201       GAGCGGGTGCGGCTCGTGACCAGACTCATCTACAACCGGGAGGAGTTCGT
Eqze-DQB3*0101       GAGCGGGTGCGGCTCGTGACCAGACTCATCTACAACCGGGAGGAGTTCGT
Eqbu-DQB3*0101       GAGCGGGTGCGGCTCGTGACCAGACTCATCTACAACCGGGAGGAGTTCGT
Eqbu-DQB3*0201       GAGCGGGTGCGGCTCTTGACCAGACTCATCTACAACCGGGAGGAGTTCGT
Eqbu-DQB3*0301       GAGCGGGTGCGGCTCGTGACCAGACTCATCTACAACCGGGAGGAGTTCGT
Eqbu-DQB3*0401       GAGCGGGTGCGGCTCGTGACCAGACTCATCTACAACCGGGAGGAGTTCGT
Eqas-DQB3*0101       GAGCGGGTGCGGCTCGTGACCAGACTCATCTACAACCGGGAGGAGTTCGT
Eqas-DQB3*0201       GAGCGGGTGCGGCTCGTGACCAGACTCATCTACAACCGGGAGGAGTTCGT
Eqas-DQB3*0301       GAGCGGGTGCGGCTCGTGACCAGACTCATCTACAACCGGGAGGAGTTCGT
Eqhe-DQB3*0101       GAGCGGGTGCGGCTCGTGACCAGACTCGTCTACAACCGGGAGGAGTTCGT
Eqhe-DQB3*0201       GAGCGGGTGCGGCTCGTGACCAGACTCATCTACAACCGGGAGGAGTTCGT
Eqki-DQB3*0101       GAGCGGGTGCGGCTCGTGACCAGACTCATCTACAACCGGGAGGAGTTCGT
Eqki-DQB3*0201       GAGCGGGTGCGGCTCGTGACCAGACTCATCTACAACCGGGAGGAGTTCGT
Eqki-DQB3*0301       GAGCGGGTGCGGCTCGTGACCAGACTCATCTACAACCGGGAGGAGTTCGT
Eqca-DQBPr*0101      GAGCGGGTGCGGCTCGTGACCAGACTCATCTATAACCTGGAGGAGTACGC
Eqze-DQBPr*0101      GAGCGGGTGCGGCTCGTGACCAGACTCATCTATAACCTGGAGGAGTACGC
Eqbu-DQBPr*0101      GAGCGGGTGCGGCTCGTGACCAGACTCATCTATAACCTGGAGGAGTACGC
Eqbu-DQBPr*0201      GAGCGGGTGCGGCTCGTGACCAGACTCATCTATAACCTGGAGGAGTACGC
Eqbu-DQBPr*0301      GAGCGGGTGCGGCTCGTGACCAGACTCATCTATAACCTGGAGGAGTACGC
Eqbu-DQBPr*0401      GAGCGGGTGCGGCTCGTGACCAGACTCATCTATAACCTGGAGGAGTACGC
Eqas-DQBPr*0101      GAGCGGGTGCGGCTCGTGACCAGATACATCTACAACCTGGAGGAGTACGC
Eqas-DQBPr*0201      GAGCGGGTGCGGCTCGTGACCAGACTCATCTATAACCTGGAGGAGTACGC
Eqhe-DQBPr*0101      GAGCGGGTGCGGCTCGTGACCAGACTCATCTATAACCTGGAGGAGTACGC
Eqhe-DQBPr*0201      GAGCGGGTGCGGCTCGTGACCAGATACATCTACAACCTGGAGGAGTACGC
Eqki-DQBPr*0101      GAGCGGGTGCGGCTCGTGACCAGACTCATCTATAACCTGGAGGAGTACGC

                              110       120       130       140       150
                     ....|....|....|....|....|....|....|....|....|....|
Eqca-DQB1*00101      GCGCTTCGACAGCGACGTGGGGGAGTACCGGGCGCTGACCGAGCAGGGGC
(Eqca-DQB1*00201)    GCGCTTCGACAGCGACGTGGGGGAGTACCGGGCGCTGACCGAGCTGGGGC
(Eqca-DQB1*00301)    GCGCTTCGACAGCGACGTGGGGGAGTACCGGGCGCTGACCGAGCTGGGGC
(Eqca-DQB1*00401)    GCGCTTTGACAGCGACGTGGGGGAGTTCCGGGCGCTGACCGAGCTGGGGC
Eqca-DQB1*00501      GCGCTTCGACAGCGACGTGGGGGAGTTCCGGGCGCTGACCGAGCTGGGGC
Eqca-DQB1*0601       GCGCTTAGACAGCGACGTGGGGGAGTTCCGGGCGCTGACCCAGCTGGGGC
Eqca-DQB1*0701       GCGCTTCGACAGCGACGTGGGGGAGTACCGGGCGCTGACCGAGCAGGGGC
Eqca-DQB1*0801       GCGCTTAGACAGCGACGTGGGGGAGTTCCGGGCGCTGACCCAGCTGGGGC
Eqpr-DQB1*0101       GCGCTTCGACAGCGACGTGGGGGAGTTCCGGGCGGTGACCGAGCTGGGGC
Eqgr-DQB1*0101       GCGCTTCGACAGCGACGTGGGGGAGTTCCGGGCGGTGACCGAGCTGGGGC
Eqze-DQB1*0101       GCGCTTCGACAGCGACGTGGGGGAGTTCCGGGCGGTGACCGAGCTGGGGC
Eqbu-DQB1*0101       GCGCTTCGACAGCGACGTGGGGGAGTTCCGGGCGGTGACCGAGCTGGGGC
Eqbu-DQB1*0201       GCGCTTCGACAGCGACGTGGGGGAGTTCCGGGCGCTGACCGAGCTGGGGC
Eqbu-DQB1*0301       GCGCTTCGACAGCGACGTGGGGGAGTACCGGGCGCTGACCGAGCAGGGGC
Eqbu-DQB1*0401       GCGCTTCGACAGCGACGTGGGGGAGTACCGGGCGCTGACCGAGCAGGGGC
Eqbu-DQB1*0501       GCGCTTCGACAGCGACGTGGGGGAGTACCTGGCGCTGACCCAGCTGGGGC
Eqas-DQB1*0101       GCGCTTCGACAGCGACGTGGGGGAGTACCGGGCGGTGACCGAGCTGGGGC
Eqhe-DQB1*0101       GCGCTTCGACAGCGACGTGGGGGAGTTCCGGGCGGTGACCGAGCTGGGGC
Eqhe-DQB1*0201       GCGCTTCGACAGCGACGTGGGGGAGTTCCGGGCGCTGACCGAGCTGGGGC
Eqki-DQB1*0101       GCGCTTCGACAGCGACGTGGGGGAGTTCCGGGCGGTGACCGAGCTGGGGC
Eqki-DQB1*0201       GCGCTTCGACAGCGACGTGGGGGAGTTCCGGGCGGTGACCGAGCTGGGGC
Eqca-DQB2*00101      GCGCTTCGACAGCGACGTGGGGGAGTACCGGGCGCTGACCGAGCTGGGAC
                              110       120       130       140       150
```

```
                        ....|....|....|....|....|....|....|....|....|....|
(Eqca-DQB2*00201)       GCGCTTCGACAGCGACGTGGGGGAGTACAAGGCGGTGACCGAGCTGGGGC
(Eqca-DQB2*00301)       GCGCTTCGACAGCGACGTGGGGGTGTACCAGGCGGTGACCGAGCTGGGGC
Eqca-DQB2*00401         GCGCTTCGACAGCGACGTGGGGGAGTACCGGGCGCTGACCGAGGCGGGGC
(Eqca-DQB2*00501)       GCGCTTCGACAGCGACGTGGGGGAGTACCAAGCGGTGACCGAGCTGGGGC
Eqca-DQB2*0601          GCGCTTCGACAGCGACGTGGGGGAGTACCGGGCGCTGACCGAGGCGGGGC
Eqca-DQB2*0701          GCGCTTCGACAGCGACGTGGGGGAGTACCGGGCGCTGACCGAGGCGGGGC
Eqca-DQB2*0801          GCGCTTCGACAGCGACGTGGGGGAGTACCGGGCGGTGACCGAGCTGGGGC
Eqpr-DQB2*0101          GCGCTTCGACAGCGACGTGGGGGAGTACCGGGCGGTGACCGAGCTGGGGC
Eqpr-DQB2*0201          GCGGTTCGACAGCGACGTGGGGGAGTACCGGGCGGTGACCGAGCTGGGGC
Eqgr-DQB2*0101          GCGCTTCGACAGCGACGTGGGGGAGTACCGGGCGCTGACCGAGGCTGGGC
Eqze-DQB2*0101          GCGCTTCGACAGCGACGTGGGGGAGTACCGGGCGCTGACCGAGGCGGGGC
Eqbu-DQB2*0101          GCGCTTCGACAGCGACGTGGGGGAGTACCGGGCGCTGACCGAGCTGGGAC
Eqbu-DQB2*0201          GCGCTTCGACAGCGACGTGGGGGAGTACCGGGCGCTGACCGAGGCGGGGC
Eqbu-DQB2*0301          GCGCTTCGACAGCGACGTGGGGGAGTACCGGGCGCTGACCGAGCTGGGAC
Eqbu-DQB2*0401          GCGCTTCGACAGCGACGTGGGGGAGTACCGGGCGCTGACCGAGGCGGGGC
Eqas-DQB2*0101          GCGCTTCGACAGCGACGTGGGGGAGTACCGGGCGCTGACCGAGGCGGGGC
Eqas-DQB2*0201          GCGCTTCGACAGCGACGTGGGGGAGTACCGGGCGCTGACCGAGGCGGGGC
Eqas-DQB2*0301          GCGCTTCGACAGCGACGTGGGGGAGTACCGGGCGCTGACCGAGGCGGGGC
Eqki-DQB2*0101          GCGCTTCGACAGCGACGTGGGGGAGTACCGGGCGCTGACCGAGCTGGGAC
Eqca-DQB3*00101         GCGCTTCGACAGCGACGTGGGGGAGTTCCAGGCAGTGACCGAGCTGGGGC
(Eqca-DQB3*00201)       GCGCTTCGACAGCGACGTGGGGGAGTTCCAGGCAGTGACCGAGCTGGGGC
Eqca-DQB3*0301          GCGCTTCGACAGCGACGTGGGGGAGTTCCAGGCAGTGACCGAGCTGGGGC
Eqca-DQB3*0401          GCGCTTCGACAGCGACGTGGGGGAGTTCCAGGCAGTGACCGAGCTGGGGC
Eqca-DQB3*0501          GCGCTTCGACAGCGACGTGGGGGAGTTCCAGGCAGTGACCGAGCTGGGGC
Eqpr-DQB3*0101          GCGCTTCGACAGCGACGTGGGGGAGTTCCAGGCAGTGACCGAGCTGGGGC
Eqpr-DQB3*0201          GCGCTTCGACAGCGACGTGGGGGAGTTCCAGGCAGTGACCGAGCTGGGGC
Eqpr-DQB3*0301          GCGCTTCGACAGCGACGTGGGGGAGTTCCAGGCAGTGACCGAGCTGGGGC
Eqpr-DQB3*0401          GCGCTTCGACAGCGACGTGGGGGAGTTCCAGGCAGTGACCGAGCTGGGGC
Eqgr-DQB3*0101          GCGCTTCGACAGCGACGTGGGGGAGTTCCAGGCAGTGACCGAGCTGGGGC
Eqgr-DQB3*0201          GCGCTTCGACAGCGACGTGGGGGAGTTCCAGGCAGTGACCGAGCTGGGGC
Eqze-DQB3*0101          GCGCTTCGACAGCGACGTGGGGGAGTTCCAGGCAGTGACCGAGCTGGGGC
Eqbu-DQB3*0101          GCGCTTCGACAGCGACGTGGGGGAGTTCCAGGCAGTGACCGAGCTGGGGC
Eqbu-DQB3*0201          GCGCTTCGACAGCGACGTGGGGGAGTTCCAGGCAGTGACTGAGCTGGGGC
Eqbu-DQB3*0301          GCGCTTCGACAGCGACGTGGGGGAGTTCCAGGCAGTGACCGAGCTGGGGC
Eqbu-DQB3*0401          GCGCTTCGACAGCGACGTGGGGGAGTTCCAGGCAGTGACCGAGCTGGGGC
Eqas-DQB3*0101          GCGCTTCGACAGCGACGTGGGGGAGTTCCAGGCAGTGACCGAGCTGGGGC
Eqas-DQB3*0201          GCGCTTCGACAGCGACGTGGGGGAGTTCCAGGCAGTGACCGAGCTGGGGC
Eqas-DQB3*0301          GCGCTTCGACAGCGACGTGGGGGAGTTCCAGGCAGTGACCGAGCTGGGGC
Eqhe-DQB3*0101          GCGCTTCGACAGCGACGTGGGGGAGTTCCAGGCAGTGACCGAGCTGGGGC
Eqhe-DQB3*0201          GCGCTTCGACAGCGACGTGGGGGAGTTCCAGGCAGTGACCGAGCTGGGGC
Eqki-DQB3*0101          GCGCTTCGACAGCGACGTGGGGGAGTTCCAGGCAGTGACCGAGCTGGGGC
Eqki-DQB3*0201          GCGCTTCGACAGCGACGTGGGGGAGTTCCAGGCAGTGACCGAGCTGGGGC
Eqki-DQB3*0301          GCGCTTCGACAGCGACGTGGGGGAGTTCCAGGCAGTGACCGAGCTGGGGC
Eqca-DQBPr*0101         GCGCTTCGACAGCGACGTGGGGGTGTACCAGGCGGTGACCGAGCTGGGGC
Eqze-DQBPr*0101         GCGCTTCGACAGCGACGTGGGGGTGTACCAGGCGGTGACCGAGCTGGGGC
Eqbu-DQBPr*0101         GCGCTTCGACAGCGACGTGGGGGTGTACCAGGCGGTGACCGAGCTGGGGC
Eqbu-DQBPr*0201         GCGCTTCGACAGCGACGTGGGGGTGTACCAGGCGGTGACCGAGCTGGGGC
Eqbu-DQBPr*0301         GCGCTTCGACAGCGACGTGGGGGTGTACCAGGCGGTGACCGAGCTGGGGC
Eqbu-DQBPr*0401         GCGCTTCGACAGCGACGTGGGGGTGTACCAGGCGGTGACCGAGCTGGGGC
Eqas-DQBPr*0101         GCGCTTCGACAGCGACGTGGGGGAGTACCAGGCTGTAACCGAGCTGGGGC
Eqas-DQBPr*0201         GCGCTTCGACAGCGACGTGGGGGTGTACCAGGCGGTGACCGAGCTGGGGC
Eqhe-DQBPr*0101         GCGCTTCGACAGCGACGTGGGGGTGTACCAGGCGGTGACCGAGCTGGGGC
Eqhe-DQBPr*0201         GCGCTTCGACAGCGACGTGGGGGAGTACCGGGCGGTGACCGAGCTGGCGC
Eqki-DQBPr*0101         GCGCTTCGACAGCGACGTGGGGGTGTACCAGGCGGTGACCGAGCTGGGGC

                                  160       170       180       190       200
```

```
                     ....|....|....|....|....|....|....|....|....|....|
Eqca-DQB1*00101      GGCCGGACGCCGAGGACTGGAACGGGCAGAAGGACTTCCTGGAGCAGACG
(Eqca-DQB1*00201)    GGCCGACCGCCGAGTACTGGAACGGGCAGAAGGACGTCCTGGAGCGGACG
(Eqca-DQB1*00301)    GGCACATTGCCGAGGACTGGAACGGGCAGAAGGACGTCCTGGAGCAGACG
(Eqca-DQB1*00401)    GGCCGGGAGCCGAGGCCTGGAACCAACAGCAGGACTTCCTGGAGCAGTAC
Eqca-DQB1*00501      GGCCAGACGCCGAGTACTGGAACGGGCAGAAGGACACCCTGGAGGAGTAC
Eqca-DQB1*0601       GGTGCATTGCCTAGGACTGGAACGGGCAGAAGGACGTCCTGGAGCGGGTG
Eqca-DQB1*0701       GGCCGGACGCCGAGTACTGGAACGGGCAGAAGGACATCCTGGAGCAGACG
Eqca-DQB1*0801       GGTGCATTGCCTAGGACTGGAACGGGCAGAAGGACGTCCTGGAGCGGGTG
Eqpr-DQB1*0101       GGCCGGACGCCGAGTACTGGAACGGGCAGAAGGACTTCCTGGAGGAGTAC
Eqgr-DQB1*0101       GGCCGGACGCCGAGTACTGGAACGGGCAGAAGGACTTCCTGGAGGAGTAC
Eqze-DQB1*0101       GGCCGGACGCCGAGTACTGGAACGGGCAGAAGGACTTCCTGGAGGAGTAC
Eqbu-DQB1*0101       GGCCGGACGCCGAGTACTGGAACGGGCAGAAGGACTTCCTGGAGGAGTAC
Eqbu-DQB1*0201       GGCCGGACGCCGAGTACTGGAACGGGCAGAAGGACCTCATGGAGCAGCAC
Eqbu-DQB1*0301       GGCCGGACACCGAGTACTGGAACGGGCAGAAAGACCTCCTGGAGCGCAAC
Eqbu-DQB1*0401       GGCCGGACACCGAGTACTGGAACGGGCAGAAGGACCTCCTGGAGCAGACG
Eqbu-DQB1*0501       GGCACATTGCCGAGGACTGGAACGGGCAGGAGGACCTCCTGGAGCAGGAC
Eqas-DQB1*0101       GGCGGCAGGCCGAGTACTGGAACACGCAGAAGGACGAACTGGAGCGGGTG
Eqhe-DQB1*0101       GGCCGGACGCCGAGTACTGGAACGGGCAGAAGGACTTCCTGGAGGAGTAC
Eqhe-DQB1*0201       GGCCGGACGCCGAGTACTGGAACGGGCAGAAGGACTTCCTGGAGCAGACG
Eqki-DQB1*0101       GGCACATTGCCGAGGACTGGAACGGGCAGGACGACCTCCTGGAGCGGGTG
Eqki-DQB1*0201       GGCCGGACGCCGAGTACTGGAACGGGCAGAGGGACGTCCTGGAGCGGGTG
Eqca-DQB2*00101      GGCCGGAAGCCGAGTACTGGAACGGACAGAAGGACATCCTGGAGGGGACG
(Eqca-DQB2*00201)    GGCCGAGCGCCGAGTACTGGAACGGACAGAAGGACGTCCTGGAGCAGACG
(Eqca-DQB2*00301)    GGCCGTGCACCGAGTACTGGAACGGGCAGAAGGACGAACTGGAACGGGTG
Eqca-DQB2*00401      GGCCGGCAGCCGAGTACTGGAACGGGCAGAAGGACATCCTGGAGAAGACG
(Eqca-DQB2*00501)    GGCGGAGCGCCGAGTACTGGAACGGGCAGAAGGACGTCCTGGATGACGCG
Eqca-DQB2*0601       GGCCGGACGCCGAGTACTGGAACGGACAGAAGGACATCCTGGAGCGGACG
Eqca-DQB2*0701       GGCCGGACGCCGAGTACTGGAACGGGCAGAAGGACTTCCTGGAGCAGACG
Eqca-DQB2*0801       AGCCGGACGCCGAGTACTGGAACGGGCAGAAGGACATCCTGGAGAAGACG
Eqpr-DQB2*0101       AGCCGGACGCCGAGTACTGGAACGGGCAGAAGGACATCCTGGAGAAGACG
Eqpr-DQB2*0201       GGCCGGCAGCCGAGTACTGGAACGGGCAGAAGGACATCCTGGAGCGGACG
Eqgr-DQB2*0101       GGCCGGACGCCGAGTACTGGAACGGACAGAAGGACATCCTGGAGAGGACG
Eqze-DQB2*0101       GGCCGGACGCCGAGTACTGGAACGGACAGAAGGACATCCTGGAGGGGACG
Eqbu-DQB2*0101       GGCCGGAAGCCGAGTACTGGAACGGGCAGAAGGACATCCTGGAGGGGACG
Eqbu-DQB2*0201       GGCCGGCAGCCGAGTACTGGAACGGACAGAAGGACATCCTGGAGAGGACG
Eqbu-DQB2*0301       GGCCGGCAGCCGAGTACTGGAACGGACAGAAGGACATCCTGGAGGGGACG
Eqbu-DQB2*0401       GGCCGGACGCCGAGTACTGGAACGGACAGAAGGACATCCTGGAGAGGACG
Eqas-DQB2*0101       GGCCGGCAGCCGAGTACTGGAACGGACAGAAGGACATCCTGGAGAGGACG
Eqas-DQB2*0201       GGCCGGCAGCCGAGTACTGGAACGGACAGAAGGACATCCTGGAGAGGACG
Eqas-DQB2*0301       GGCCGGCAGCCGAGTACTGGAACGGACAGAAGGACATCCTGGAGAGGACG
Eqki-DQB2*0101       GGCCGGACGCCGAGTACTGGAACGGACAGAAGGACATCCTGGAGGGGACG
Eqca-DQB3*00101      GGCACATTGCCGAGGACTGGAACGGACAGAAGGACGTCCTGGAGCAGAAG
(Eqca-DQB3*00201)    GGCACATTGCCGAGGACTGGAACGGACAGAAGGACGTCCTGGAGCAGAAG
Eqca-DQB3*0301       GGCACATTGCCGAGGACTGGAACGGACAGAAGGACGTCCTGGAGCAGAAG
Eqca-DQB3*0401       GGCACATTGCCGAGGACTGGAACGGACAGAAGGACGTCCTGGAGCAGAAG
Eqca-DQB3*0501       GGCACATTGCCGAGGACTGGAACGGACAGAAGGACGTCCTGGAGCAGAAG
Eqpr-DQB3*0101       GGCACATTGCCGAGGACTGGAACGGACAGAAGGACGTCCTGGAGCAGAAG
Eqpr-DQB3*0201       GGCACATTGCCAAGGACTGGAACGGACAGAAGGACGTCCTGGAGCAGAAG
Eqpr-DQB3*0301       GGCACATTGCCGAGGACTGGAACGGACAGAAGGACGTCCTGGAGCAGAAG
Eqpr-DQB3*0401       GGCACATTGCCGAGGACTGGAACGGACAGAGGGACGTCCTGGAGCAGAAG
Eqgr-DQB3*0101       GGCACATTGCCGAGGACTGGAACGGACAGAAGGACGTCCTGGAGCAGAAG
Eqgr-DQB3*0201       GGCACATTGCCGAGGACTGGAACGGACAGAAGGACGTCCTGGAGCAGAAG
Eqze-DQB3*0101       GGCACATTGCCGAGGACTGGAACGGACAGAAGGACGTCCTGGAGCAGAAG
Eqbu-DQB3*0101       GGCACATTGCCGAGGACTGGAACGGACAGAAGGACGTCCTGGAGCAGAAG
Eqbu-DQB3*0201       GGCACATTGCCGAGGACTGGAACAGACAGAAGGACGTCCTGGAGCAGAAG
Eqbu-DQB3*0301       GGCACATTGCCAAGGACTGGAACGGACAGAAGGACGTCCTGGAGCAGAAG
Eqbu-DQB3*0401       GGCACATTGCCGAGGACTGGAATGGACAGAAGGACGTCCTGGAGCAGAAG
Eqas-DQB3*0101       GGCACATTGCCGAGGACTGGAACGGACAGAAGGACGTCCTGGAGCAGAAG
Eqas-DQB3*0201       GGCACATTGCCAAGGACTGGAACGGACAGAAGGACGTCCTGGAGCAGAAG
Eqas-DQB3*0301       GGCACATTGCCGAGGACTGGAACAGACAGAAGGACGTCCTGGAGCAGAAG
                              160       170       180       190       200
```

```
                        ....|....|....|....|....|....|....|....|....|....|
Eqhe-DQB3*0101          GGCACATTGCCGAGGACTGGAACAGACAGAAGGACGTCCTGGAGCAGAAG
Eqhe-DQB3*0201          GGCACATTGCCAAGGACTGGAACGGACAGAAGGACGTCCTGGAGCAGAAG
Eqki-DQB3*0101          GGCACATTGCCGAGGACTGGAACGGACAGAAGGACGTCCTGGAGCAGAAG
Eqki-DQB3*0201          GGCACATTGCCGAGGACTGGAACAGACAGAAGGACGTCCTGGAGCAGAAG
Eqki-DQB3*0301          GGCACATTCCCGAGGACTGGAACGGACAGAAGGACGTCCTGGAGCAGAAG
Eqca-DQBPr*0101         GGCCGTGCACCGAGTACTGGAACGGGCAGAAGGACGAACTGGAACGGGTG
Eqze-DQBPr*0101         GGTGGAGCACCGAGTACTGGAACGGGCAGAAGGACGAACTGGAACGGGTG
Eqbu-DQBPr*0101         GGTGGAGCACCGAGTACTGGAACGGGCAGAAGGACGAACTGGAACGGGTG
Eqbu-DQBPr*0201         GGTGGAGCACCGAGTACTGGAACGGGCAGAAGGACGAACTGGAACGGGTG
Eqbu-DQBPr*0301         GGTGGAGCACCGAGTACTGGAACGGGCAGAAGGACGAACTGGAACGGGTG
Eqbu-DQBPr*0401         GGTGGAGCACCGAGTACTGGAACGGGCAGAAGGACGAACTGGAACGGGTG
Eqas-DQBPr*0101         GGCTGGACGCCGAGTACTGGAACGGACAGAAGGACGTCCTGGAGCAGACG
Eqas-DQBPr*0201         GGCCGAGCACCGAGTACTGGAACGGGCAGAAGGACGAACTGGAACGGGTG
Eqhe-DQBPr*0101         GGTCGAGCACCGAGTACTGGAACGGGCAGAAGGACGAACTGGAACGGGTG
Eqhe-DQBPr*0201         GGCGGAGCGCCGAGTACTGGAACGGACAGAAGGACGTCCTGGAGCAGACG
Eqki-DQBPr*0101         GGTGGAGCACCGAGTACTGGAACGGGCAGAAGGACGAACTGGAACGGGTG

                                  210       220       230       240       250
                        ....|....|....|....|....|....|....|....|....|....|
Eqca-DQB1*00101         CGGGCCTACGTGGACACGGTGTGCAGACACAACTACCAGGTGGAGGCCCC
(Eqca-DQB1*00201)       CGGGCCGAGATAGACACGGTGTGCAGACACAACTACCAGCTGGAGGCCCT
(Eqca-DQB1*00301)       CGGGCCGAGACGGACACGGTGTGCAGACACAACTACCAGCTGGAGGCCCT
(Eqca-DQB1*00401)       CGGGCCTACGTGGACACGTTGTGCAGACACAACTACCAGCTGGAGGCCCT
Eqca-DQB1*00501         CGGGCCTACGTGGACACGTTGTGCAGACACAACTACCAGATAGAGGTCCT
Eqca-DQB1*0601          CAGGCCGACGTGGACACGGTGTGCAGACACAACTACGAGGGGTGGGGGGC
Eqca-DQB1*0701          CGGGCCGAGGTGGACACGGTGTGCAGACACAACTACCAGACGGAGGTCCC
Eqca-DQB1*0801          CAGGCCGACGTGGACACGGTGTGCAGACACAACTACGAGGGGGGGGGGCC
Eqpr-DQB1*0101          CGGGCCTACGTGGACACGTTGTGCAGACACAACTACCAGATAGAGGTCCT
Eqgr-DQB1*0101          CGGGCCTACGTGGACACGTTGTGCAGACACAACTACCAGATAGAGGTCCT
Eqze-DQB1*0101          CGGGCCTACGTGGACACGTTGTGCAGACACAACTACCAGATAGAGGTCCT
Eqbu-DQB1*0101          CGGGCCTACGTGGACACGTTGTGCAGACACAACTACCAGATAGAGGTCCT
Eqbu-DQB1*0201          CGGGCCTACGTGGACACGTTGTGCAGACACAACTACCAGGTGGAGGTCCT
Eqbu-DQB1*0301          CGCGCCGAGGTGGACACGGTGTGCAGACACAACTACCAGGCGTACGCCCC
Eqbu-DQB1*0401          CGGGCCGAGGTGGACAGGGTGTGCAGACACAACTACCAGGCGTACGCCCC
Eqbu-DQB1*0501          CGCGCCGCGGTGGACACGGTGTGCAGACACAACTACGAGGTGGACGCCCC
Eqas-DQB1*0101          CGGGCTGAGGTGGACACGGTGTGCAGACACAACTACCAGGTGGACGCCCC
Eqhe-DQB1*0101          CGGGCCTACGTGGACACGTTGTGCAGACACAACTACCAGATAGAGGTCCT
Eqhe-DQB1*0201          CGGGCCGCGGTGGACACGGTGTGCAGACACAACTACCAGGTGGAGGCCCT
Eqki-DQB1*0101          CGGGCCGAGGTGGACAAGGTGTGCAGACACAACTACCAGGTGTACGCCCC
Eqki-DQB1*0201          CGGGCCGAGGTGGACAAGGTGTGCAGACACAACTACCAGGGGGTGGGGCC
Eqca-DQB2*00101         CGGGCCGAGCTGGACAGGGTGTGCCGACCCAACTACCAGTTGGAGTTCCC
(Eqca-DQB2*00201)       CGGGCCGAGTTGGACAGAGTGTGCAGATACAACTACCAGTTGGAGGTCCC
(Eqca-DQB2*00301)       CGGGCCGGGGTGGACCGTGTGTGCAGACACAACTACAAGTTGGAGGTCCC
Eqca-DQB2*00401         TGGGCCGAGGTGGACAGGGTGTGCAGAAACAACTACCAGGTGGAGGCCCC
(Eqca-DQB2*00501)       CGGGCCGCGGTGGACACGGTGTGCAGATACAACTACCAGTTGGAGGTCCC
Eqca-DQB2*0601          CGGGCCGAGTTGGACAGGGTGTGCAGAAACAACTACCAGGTGGATGCCCC
Eqca-DQB2*0701          CGGGCCGAACTGGACACGGTGTGCAGACACAACTACCAGGTGGAGGCCCC
Eqca-DQB2*0801          TGGGCCGAGGTGGACAGGGTGTGCAGAAACAACTACCAGGTGGAGGCCCC
Eqpr-DQB2*0101          TGGGCCGAGGTGGACAGGGTGTGCAGAAACAACTACCAGGTGGAGGCCCC
Eqpr-DQB2*0201          CGGGCCGAGTTGGACAGGGTGTGCAGAAACAACTACCAGGTGGAGGCCCC
Eqgr-DQB2*0101          CGGGCCGAGCTGGACAGGGTGTGCCGACCCAACTACCAGTTGGAGTTCCC
Eqze-DQB2*0101          CGGGCCGAGCTGGACAGGGTGTGCCGACCCAACTACCAGTTGGAGTTCCC
Eqbu-DQB2*0101          CGGGCCGAGGTGGACAGGGTGTGCCGACACAACTACCAGTTGGAGTTCCT
Eqbu-DQB2*0201          TGGGCCGAGGTGGACAGGGTGTGCAGAAACAACTACCAGGTGGAGGCCCC
Eqbu-DQB2*0301          CGGGCCGAGCTGGACAGGGTGTGCCGACACAACTACCAGACGGAGGTCCC
Eqbu-DQB2*0401          CGGGCCGAGGTGGACAGGGTGTGCAGACACAACTACCAGGTGGAGGCCCC
Eqas-DQB2*0101          TGGGCCGAGGTGGACAGGGTGTGCAGAAACAACTACCAGGTGGACGCCCC
Eqas-DQB2*0201          TGGGCCGAGGTGGACAGGGTGTGTAGAAACAACTACCAGGTGGGGGCCCC
Eqas-DQB2*0301          TGGGCCGAGGTGGACAGGGTGTGCAGAAACAACTACCAGGTGGACGCCCC
Eqki-DQB2*0101          CGGGCCGAGCTGGACAGGGTGTGCCGACACAACTACCAGTTGGAGTTCCC
                                  210       220       230       240       250
```

```
                        ....|....|....|....|....|....|....|....|....|....|
Eqca-DQB3*00101         CGGGCCGAGCTGGACACGGTGTGCAGACACAACTACCAGGTGGAGGCCCT
(Eqca-DQB3*00201)       CGGGCCGAGCTGGACACGGTGTGCAGACACAACTACCAGGTGGAGGCCCT
Eqca-DQB3*0301          CGGGCCGAGCTGGACACGGTGTGCAGACACAACTACCAGGTGGAGGCCCT
Eqca-DQB3*0401          CGGGCCGAGCTGGACACGGTGTGCAGACACAACTACCAGGTGGAGGCCCT
Eqca-DQB3*0501          CGGGCCGAGCTGGACACGGTGTGCAGACACAACTACCAGGTGGAGGCCCT
Eqpr-DQB3*0101          CGGGCCGAGCTGGACACGGTGTGCAGACACAACTACCAGGTGGAGGCCCT
Eqpr-DQB3*0201          CGGGCCGAGCTGGACACGGTGTGCAGACACAACTACCAGGTGGAGGCCCT
Eqpr-DQB3*0301          CGGGCCGAGCTGGACACGGTGTGCAGACACAACTACCAGGTGGAGGCCCT
Eqpr-DQB3*0401          CGGGCCGAGCTGGACACGGTGTGCAGACACAACTACCAGGTGGAGGCCCT
Eqgr-DQB3*0101          CGGGCCGAGCTGGACACGGTGTGCAGACACAACTACCAGGTGGAGGCCCT
Eqgr-DQB3*0201          CGGGCCGAGCTGGACACGGTGTGCAGACACAACTACCAGGTGGAGGCCCT
Eqze-DQB3*0101          CGGGCCGAGCTGGACACGGTGTGCAGACACAACTACCAGGTGGAGGCCCT
Eqbu-DQB3*0101          CGGGCCGAGCTGGACACGGTGTGCAGACACAACTACCAGGTGGAGGCCCT
Eqbu-DQB3*0201          CGGGCCGAGCTGGACACGGTGTGCAGACACAACTACCAGGTGGAGGCCCT
Eqbu-DQB3*0301          CGGGCCGAGCTGGACACGGTGTGCAGACACAACTACCAGGTGGAGGCCCT
Eqbu-DQB3*0401          CGGGCCGAGCTGGACACGGTGTGCAGACACAACTACCAGGTGGAGGCCCT
Eqas-DQB3*0101          CGGGCCGAGCTGGACACGGTGTGCAGACACAACTACCAGGTGGAGGCCCT
Eqas-DQB3*0201          CGGGCCGAGCTGGACACGGTGTGCAGACACAACTACCAGGTGGAGGCCCT
Eqas-DQB3*0301          CGGGCCGAGCTGGACACGGTGTGCAGACACAACTACCAGGTGGAGGCCCT
Eqhe-DQB3*0101          CGGGCCGAGCTGGACACGGTGTGCAGACACAACTACCAGGTGGAGGCCCT
Eqhe-DQB3*0201          CGGGCCGAGCTGGACACGGTGTGCAGACACAACTACCAGGTGGAGGCCCT
Eqki-DQB3*0101          CGGGCCGAGCTGGACACGGTGTGCAGACACAACTACCAGGTGGAGGCCCT
Eqki-DQB3*0201          CGGGCCGAGCTGGACACGGTGTGCAGACACAACTACCAGGTGGAGGCCCT
Eqki-DQB3*0301          CGGGCCGAGCTGGACACGGTGTGCAGACACAACTACCAGGTGGAGGCCCT
Eqca-DQBPr*0101         CGGGCCGGGGTGGACCGTGTGTGCAGACACAACTACAAGTTGGAGGTCCC
Eqze-DQBPr*0101         CGGGCCGACTTGGACCGTGTGTGCAGATACAACTACCAGTTGGAGGTCCC
Eqbu-DQBPr*0101         CGGGCCGACTTGGACCGTGTGTGCAGATACAACTACCAGTTGGAGGTCCC
Eqbu-DQBPr*0201         CGGGCCGACTTGGACCGTGTGTGCAGATACAACTACCAGTTGGAGGTCCC
Eqbu-DQBPr*0301         CGGGCCGAGTTGGACCGTGTGTGCAGATACAACTACCAGTTGGAGGTCCC
Eqbu-DQBPr*0401         CGGGCCGGCTTGGACCGTGTGTGCAGACACAACTACCAGTTGGAGGTCCC
Eqas-DQBPr*0101         CGGGCCGAGTTGGACAGCGTGTGCAGACACAACTACCAGTTGGAGGTCCC
Eqas-DQBPr*0201         CGGGCCGGCGTGGACCGTGTGTGCAGACACAACTACCAGTTGGAGGTCCC
Eqhe-DQBPr*0101         CGGGCCGAGTTGGACCGTGTGTGCAGACACAACTACCAGTTGGAGGTCCC
Eqhe-DQBPr*0201         CGGGCCGAGTTGGACAGCGTGTGCAGACACAACTACCAGTTGGAGGTCCC
Eqki-DQBPr*0101         CGGGCCGACTTGGACCGTGTGTGCAGATACAACTACCAGTTGGAGGTCCC
```

```
                                      260
                        ....|....|....|....
Eqca-DQB1*00101         CTTTACCTGGCAGCGCCGA
(Eqca-DQB1*00201)       CACGACCTTACAGCGCCGA
(Eqca-DQB1*00301)       CACGACCTTACAGCGCCGA
(Eqca-DQB1*00401)       CACAAGCTTGCAGCGCCGA
Eqca-DQB1*00501         CGTGACCTTGCAGCGCCAA
Eqca-DQB1*0601          CCTTTACCGGCAGCGTCAA
Eqca-DQB1*0701          T---GCCTTGGAGCGCCGA
Eqca-DQB1*0801          CTTTACC-GGCAGCGTCAA
Eqpr-DQB1*0101          CGTGACCTTGCAGCGCCAA
Eqgr-DQB1*0101          CGTGACCTTGCAGCGCCAA
Eqze-DQB1*0101          CGTGACCTTGCAGCGCCAA
Eqbu-DQB1*0101          CGTGACCTTGCAGCGCCAA
Eqbu-DQB1*0201          CGTGACCTTGCAGCGCCAA
Eqbu-DQB1*0301          CGTTACCTGGCAGCGCCAA
Eqbu-DQB1*0401          CGTTACCTGGCAGCGCCAA
Eqbu-DQB1*0501          CTTTACCTGGCAGCGTCAA
Eqas-DQB1*0101          CTTTACCTGGCAGCGTCAA
Eqhe-DQB1*0101          CGTGACCTTGCAGCGCCAA
Eqhe-DQB1*0201          CACGACCTTGCAGCGCCGA
Eqki-DQB1*0101          CTTTACCTGGCAGCGGCAA
Eqki-DQB1*0201          CTTTACC-GGCAGCGCCAA
Eqca-DQB2*00101         C---GCCTTGGAGCGCCGA
                                      260
```

```
                      ....|....|....|....
(Eqca-DQB2*00201)     CAGGTCCTTGCAGCACCAA
(Eqca-DQB2*00301)     CAGGTCCTTGCAGCACCGA
Eqca-DQB2*00401       CTTTACCTGGCAGCGCCGA
(Eqca-DQB2*00501)     C---GCCTTGCAGCGCCAA
Eqca-DQB2*0601        CTTTACCTGGCAGCGCCAA
Eqca-DQB2*0701        CTATACCTGGCAGCGCCGA
Eqca-DQB2*0801        CTTTACCTGGCAGCGCCGA
Eqpr-DQB2*0101        CTTTACCTGGCAGCGCCGA
Eqpr-DQB2*0201        CTATACCTGGCAGCGCCAA
Eqgr-DQB2*0101        C---GCCTTGGAGCGCCGA
Eqze-DQB2*0101        C---GCCTTGGAGCGCCGA
Eqbu-DQB2*0101        C---GCCTTGGAGCGCCGA
Eqbu-DQB2*0201        CTTTACCTGGCAGCGCCGA
Eqbu-DQB2*0301        C---GCCTTGGAGCGCCGA
Eqbu-DQB2*0401        CTTTACCTGGCAGCGCCGA
Eqas-DQB2*0101        CTTTACCTGGCAGCGCCGA
Eqas-DQB2*0201        CTATACCTGGCAGCGCCGA
Eqas-DQB2*0301        CTTTACCTGGCAGCGCCGA
Eqki-DQB2*0101        C---GCCTTGGAGCGCCGA
Eqca-DQB3*00101       CACGACCTTACAGCGCCGA
(Eqca-DQB3*00201)     CACGACCTTACAGCGCCGA
Eqca-DQB3*0301        CACGACCTTACAGCGCCAA
Eqca-DQB3*0401        CACGACCTTACAGCGCCGA
Eqca-DQB3*0501        CACGACCTTACAGCGCCGA
Eqpr-DQB3*0101        CACGACCTTACAGCGCCGA
Eqpr-DQB3*0201        CACGACCTTACAGCGCCGA
Eqpr-DQB3*0301        CACGACCTTACAGCGCCGA
Eqpr-DQB3*0401        CACGACCTTACAGCGCCGA
Eqgr-DQB3*0101        CACGACCTTACAGCGCCGA
Eqgr-DQB3*0201        CACGACCTTACAGCGCCGA
Eqze-DQB3*0101        CACGACCTTACAGCGCCGA
Eqbu-DQB3*0101        CACGACCTTACAGCGCCGA
Eqbu-DQB3*0201        CACGACCTTACAGCGCCGA
Eqbu-DQB3*0301        CACGACCTTACAGCGCCGA
Eqbu-DQB3*0401        CACGACCTTACAGCGCCGA
Eqas-DQB3*0101        CACGACCTTACAGCGCCGA
Eqas-DQB3*0201        CACGACCTTACAGCGCCGA
Eqas-DQB3*0301        CACGACCTTACAGCGCCGA
Eqhe-DQB3*0101        CACGACCTTACAGCGCCGA
Eqhe-DQB3*0201        CACAACCTTACAGCGCCGA
Eqki-DQB3*0101        CACGACCTTACAGCGCCGA
Eqki-DQB3*0201        CACGACCTTACAGCGCCGA
Eqki-DQB3*0301        CACGACCTTACAGCGCCGA
Eqca-DQBPr*0101       CAGGTCCTTGCAGCACCGA
Eqze-DQBPr*0101       CAGGTCCTTGCAGCACCGA
Eqbu-DQBPr*0101       CAGGTCCTTGCAGCACCGA
Eqbu-DQBPr*0201       CAGGTCCTTGCAGCACCGA
Eqbu-DQBPr*0301       CAGGTCCTTGCAGCACCGA
Eqbu-DQBPr*0401       CAGGTCCTTGCAGCACCGA
Eqas-DQBPr*0101       CAGGTCCTTGCAGCACCGA
Eqas-DQBPr*0201       CAGGTCCTTGCAGCACCGA
Eqhe-DQBPr*0101       CAGGTCCTTGCAGCACCGA
Eqhe-DQBPr*0201       CAGGTCCTTGCAGCACCGA
Eqki-DQBPr*0101       CAGGTCCTTGCAGCACCGA
```
