# Supplementary material for: Genetic diversity, evolution and selection in the major histocompatibility complex DRB and DQB loci in the family Equidae
Source: BMC Genomics. 2020 Sep 30;21:677. doi: 10.1186/s12864-020-07089-6 (PMC7525986; doi:10.1186/s12864-020-07089-6)
Supplement: Supplementary file 4 — Additional file 4. Maximum likelihood phylogeny reconstruction of all unique DRB alleles. [file 12864_2020_7089_MOESM4_ESM.pdf]

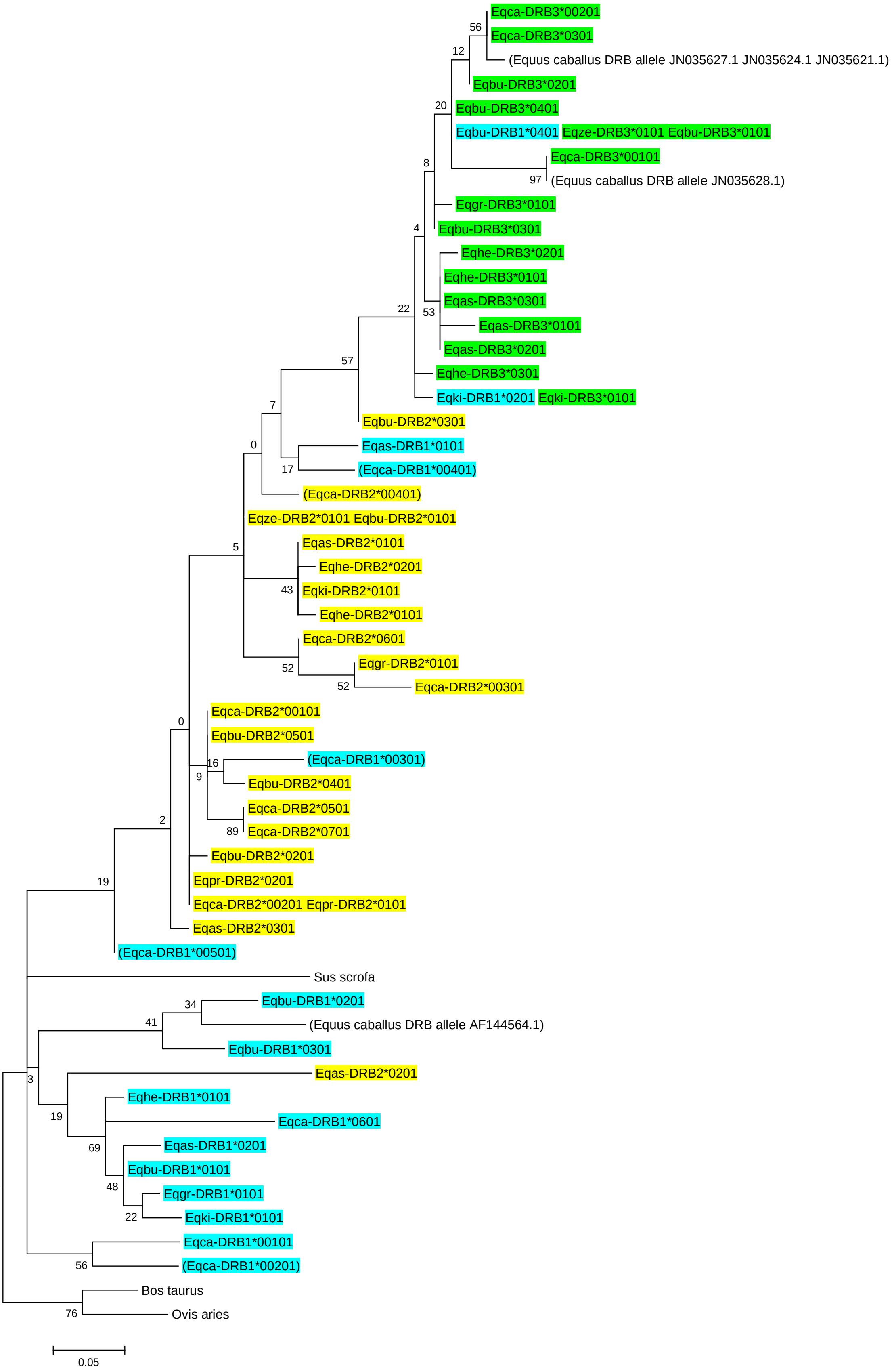

Additional file 4. **Maximum likelihood phylogeny reconstruction of all unique *DRB* alleles**. The tree was inferred using the Tamura 3-parameter model with discrete Gamma distribution and tested by 1000 Bootstrap replications. The tree is drawn to scale, with branch lengths measured in the number of substitutions per site. Sequences, which were not obtained in this study are in brackets.
