# Supplementary figures and images for: Genetic diversity, evolution and selection in the major histocompatibility complex DRB and DQB loci in the family Equidae
Source: BMC Genomics. 2020 Sep 30;21:677. doi: 10.1186/s12864-020-07089-6 (PMC7525986; doi:10.1186/s12864-020-07089-6)

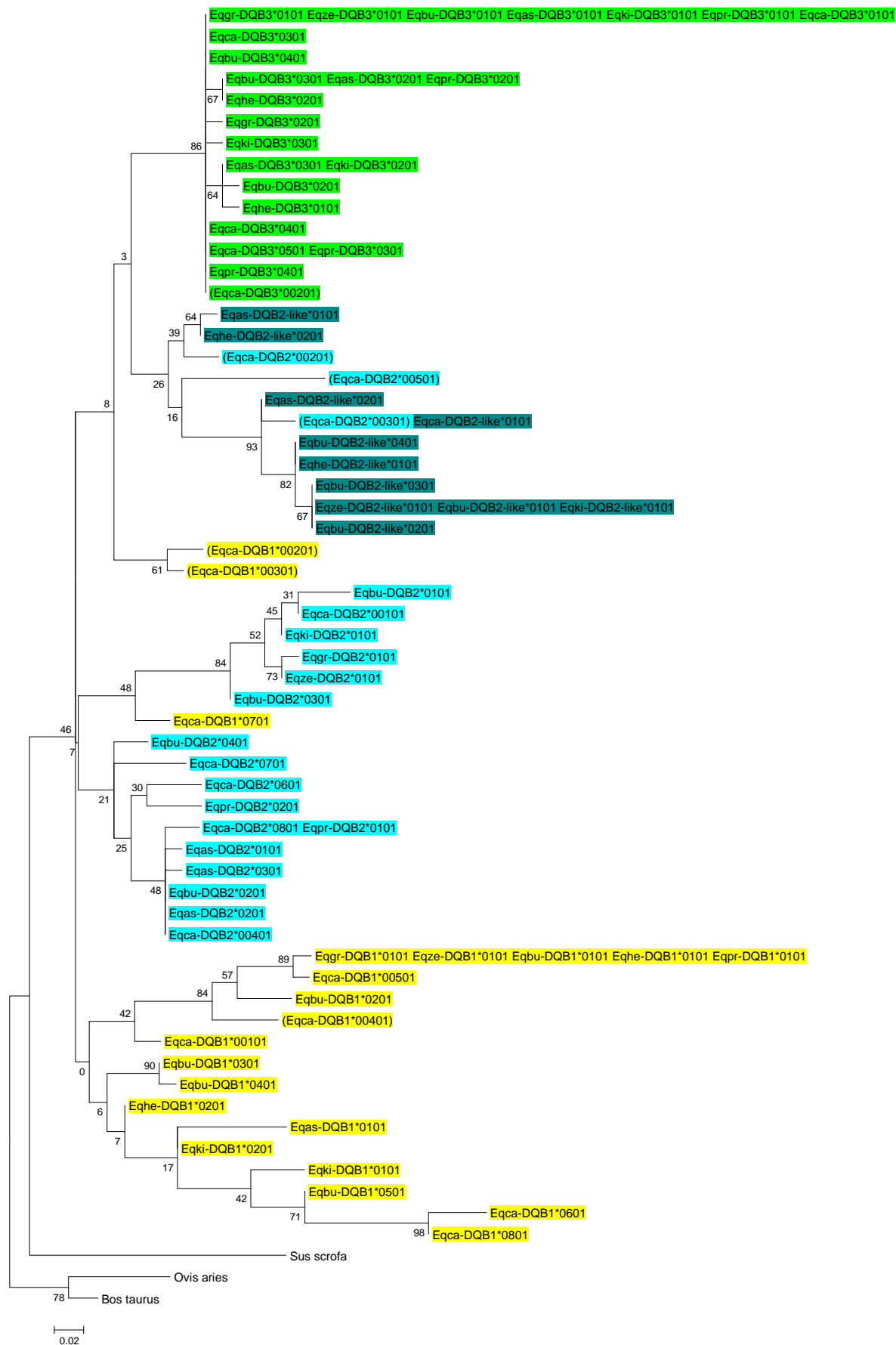

Supplement: Supplementary file 5 — Additional file 5. Maximum likelihood phylogeny reconstruction of all unique DQB alleles. The tree was inferred using the Jukes-Cantor model with discrete Gamma distribution and tested by 1000 Bootstrap replications. The tree is drawn to scale, with branch lengths measured in the number of substitutions per site. Sequences, which were not obtained in this study are in brackets. [file 12864_2020_7089_MOESM5_ESM.pdf]
