# Supplementary material for: Genetic diversity, evolution and selection in the major histocompatibility complex DRB and DQB loci in the family Equidae
Source: BMC Genomics. 2020 Sep 30;21:677. doi: 10.1186/s12864-020-07089-6 (PMC7525986; doi:10.1186/s12864-020-07089-6)
Supplement: Supplementary file 6 — Additional file 6. Maximum likelihood phylogeny reconstruction of TNFA gene. [file 12864_2020_7089_MOESM6_ESM.pdf]

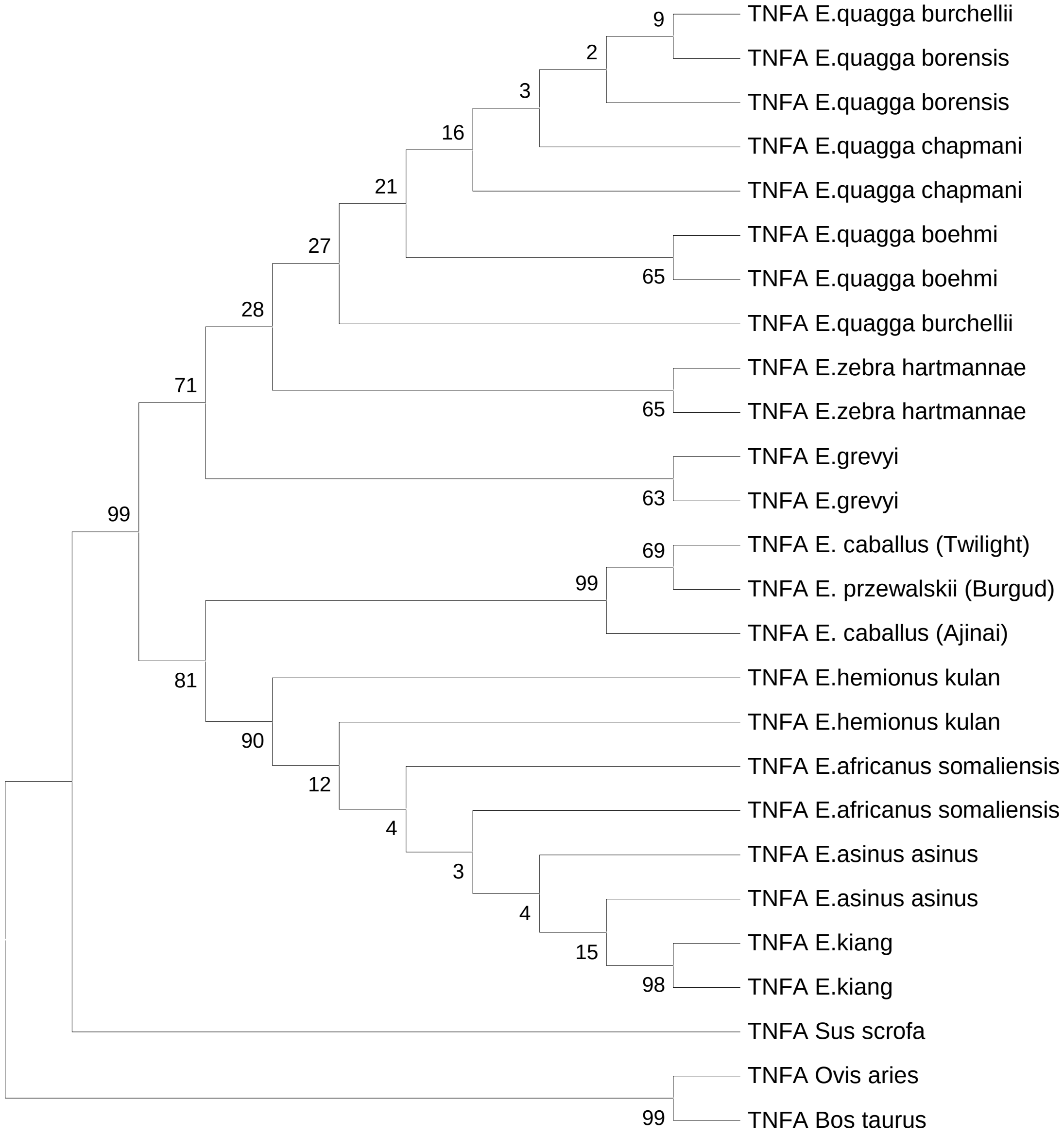

Additional file 6. **Maximum likelihood phylogeny reconstruction of *TNFA* gene**. The tree was inferred using the Hasegawa-Kishino-Yano model with discrete Gamma distribution and tested by 1000 Bootstrap replications. The tree is not drawn to scale, for better topology clarity.
